# *Hibiscus sabdariffa* extract improves hepatic steatosis, partially through IRS-1/Akt and Nrf2 signaling pathways in rats fed a high fat diet

**DOI:** 10.1038/s41598-022-11027-9

**Published:** 2022-04-29

**Authors:** Janjira Prasomthong, Nanteetip Limpeanchob, Supawadee Daodee, Pennapa Chonpathompikunlert, Sakara Tunsophon

**Affiliations:** 1 Department of Physiology, Faculty of Medical Science, Naresuan University, 99 Moo 9, Tahpoh, Muang, Phitsanulok, 65000 Thailand; 2 Department of Pharmacy Practice, Faculty of Pharmaceutical Sciences, Naresuan University, Phitsanulok, 65000 Thailand; 3 Division of Pharmaceutical Chemistry, Faculty of Pharmaceutical Sciences, Khon Kaen University, Khon Kaen, 40000 Thailand; 4 Expert Center of Innovative Health Food (Innofood), Thailand Institute of Scientific and Technological Research (TISTR), Pathumthani, 12120 Thailand; 5 Center of Excellence for Innovation in Chemistry, Naresuan University, Phitsanulok, 65000 Thailand

**Keywords:** Physiology, Diseases

## Abstract

Non-alcoholic fatty liver disease (NAFLD) has become a major world-wide health problem and is characterized by lipid accumulation in the liver induced by high fat diet (HFD) consumption. It is usually associated with inflammation, oxidative stress, and insulin resistance. Roselle extract (*Hibiscus sabdariffa*) is an herb which is used in traditional medicine. However, further study is necessary to represent the mechanism of NAFLD and find new preventive strategies. This study aims to investigate the protective effects of roselle extract on NAFLD rat models. Male Sprague–Dawley rats (n = 35) were divided into 5 groups, control, HFD, HFD + Simvastatin (HFD + SIM), HFD + 250 mg/kg BW, and HFD + 500 mg/kg BW of roselle extract (HFD + R250 and HFD + R500, respectively). The results showed that roselle extract reduced hepatic lipid contents, de novo lipogenesis enzymes, microsomal triglyceride transfer protein, inflammatory cytokines, malondialdehyde, and increased antioxidant properties, transporter related with lipoprotein uptake, and insulin signal proteins. Comparing to SIM, the HFD + R500 group exhibited the greater benefit in terms of anti-hepatic steatosis, antioxidant properties, and an ability to improve insulin resistance. This study demonstrates that roselle extract improved antioxidant properties and attenuated hepatic steatosis, liver inflammation, oxidative stress, and insulin resistance in HFD-induced NAFLD in rats, which could be used for NAFLD prevention.

## Introduction

Non-alcoholic fatty liver disease (NAFLD) is the hepatic manifestation of the metabolic syndrome and is defined as the lipid accumulation in the liver of ones who do not consume alcohol^1^. NAFLD is often related with obesity (80–90%), type 2 diabetes mellitus (30–50%) and hyperlipidemia (> 90%)^2^. The progression of NAFLD to its severe form, non-alcoholic steatohepatitis (NASH), which is characterized by fatty liver with liver inflammation and fibrosis, may lead to cirrhosis and eventually liver cell carcinoma^3^. NAFLD induced by high fat diet consumption, results in hyperlipidemia, insulin resistance, chronic inflammation and oxidative stress^4^. A high fat diet induces insulin resistance and is related to impairment of insulin signal pathways including insulin receptor substrate-1 (IRS-1)^5^.

A high fat diet is associated with oxidative stress which is defined as an imbalance between antioxidant and oxidant productions. High fat food consumption leads to the increasing levels of free fatty acids and oxidation in the liver. The by-products such as reactive oxygen species (ROS) of physiological processes including respiratory chain reaction occurring in the cells can be produced^6^. There is an increase in ROS and eventually a decrease in antioxidant capacity in hepatocytes as NAFLD progresses. Nuclear erythroid 2-related factor 2 (Nrf2) plays a crucial role in antioxidant defense mechanisms as well as anti-inflammatory response, hence the modulation of Nrf2 significantly affect the progression of NAFLD^3^. ROS serves as a pivotal regulator for inflammatory processes by modulating inflammation pathway to produce pro-inflammatory cytokines such as tumor necrosis factor and interleukin. Excessive ROS productions trigger lipid peroxidation product, and enhance the inflammatory response leading to liver damage and inflammation^7^.

Dyslipidemia is characterized by hypercholesterolemia and hypertriglyceridemia^6^. Microsomal triglyceride transfer protein (MTP) has been shown to play a central role in lipoprotein assembly by synthesis of chylomicrons and VLDL. MTP inhibitor is used to treat hyperlipidemia^7^. LDL receptor (LDLR) plays a major role in the uptake of LDL, VLDL, and chylomicron remnants which maintains lipid levels in the bloodstream^8^. LDLR and hydroxy-methylglutaryl-CoenzymeA reductase (HMGCR) have become key targets for the treatment of dyslipidemia and cardiovascular disease in recent decades^9^. Statins are drugs which are widely used for the management of hyperlipidemia, which is an HMGCR inhibitor. In patients with hypercholesterolemia, orally administered simvastatin 10–40 mg/day reduces plasma total cholesterol and LDL levels by about 30–45%^10^. On the other hand, adverse effects from statins are as follows: liver injury, statin hepatitis, hyperglycemia, renal tubule toxicity, and stroke^11–13^.

Currently, the use of alternative medicines and especially natural and herbal medicines containing phytochemicals have been rapidly rising worldwide. Roselle (*Hibiscus sabdariffa*) is a medicinal plant that has been used for decades to treat many diseases^14^. Roselle has been used in traditional medicine for its well-known medicinal properties, to promote anti-inflammatory, hepatoprotection, antioxidant, anti-hypercholesterolemia, anti-triglyceridemic and anti-hyperglycemic activities^15–18^. Especially, polyphenols and anthocyanins are of great interest due to their wide distribution in plants and help to health-promoting activities^19^. There are currently, no studies focusing on the underlying mechanism of the protective effect of roselle extract on hepatic steatosis via de novo lipogenesis protein (ACC: acetyl-CoA carboxylase, FAS: fatty acid synthase), insulin resistance, inflammation and oxidative stress in NAFLD rats which is induced by high fat diet. This study is therefore to further understand the protective effect of roselle extract on NAFLD in rats fed a high fat diet, comparing to statin.

## Results

### Phenolic compounds (HPLC analysis) in roselle extract

**Figure 1:**
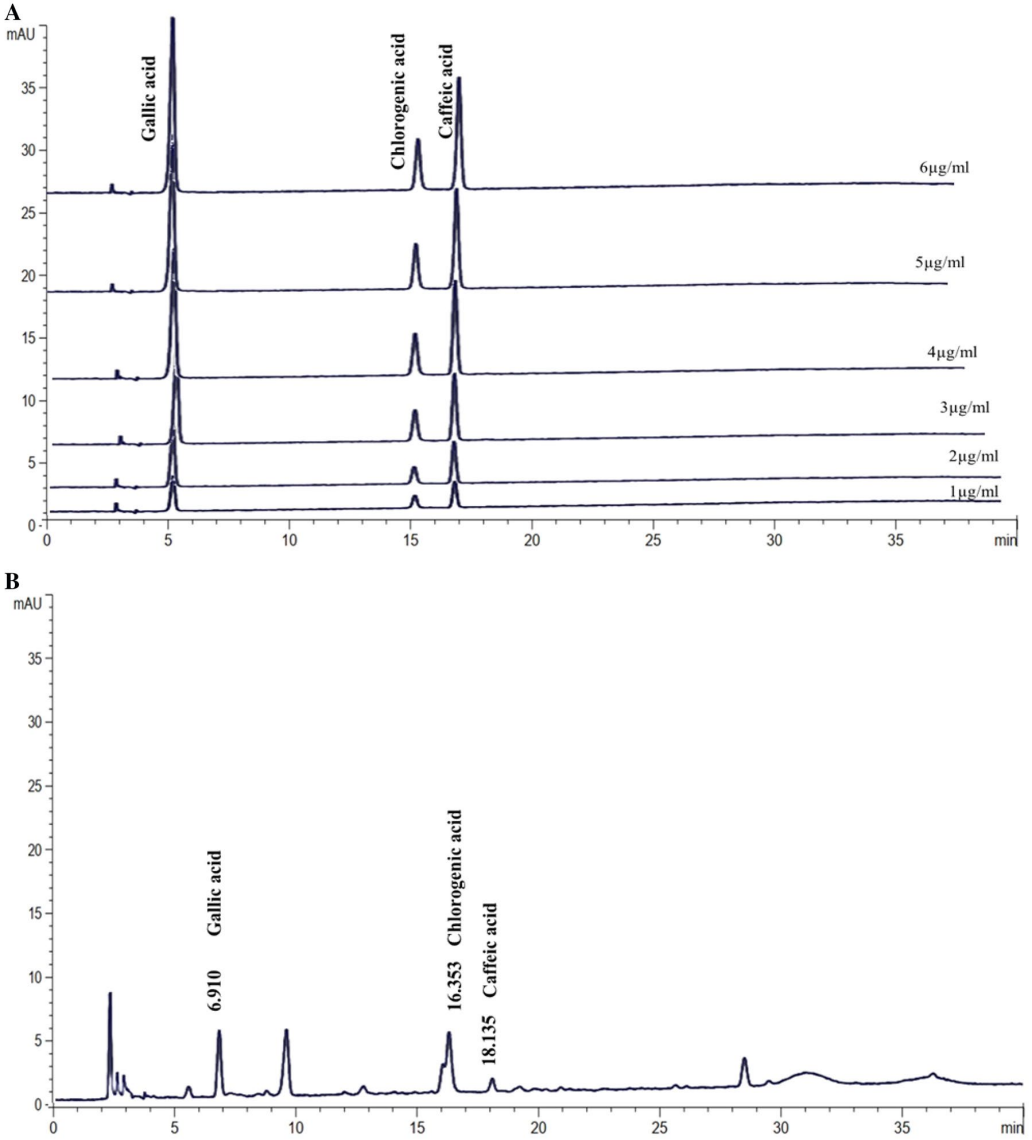
HPLC analysis of roselle extract. (**A**) HPLC chromatograms of standard gallic acid, chlorogenic acid and caffeic acid solutions at concentration 1, 2, 3, 4, 5, 6 µg/mL. (**B**) HPLC chromatogram of roselle extract at a wavelength of 280 nm.

An HPLC analysis was performed to evaluate the phenolic compounds in roselle extract (Fig. 1). Results revealed the presence of major phenolic compounds such as gallic, chlorogenic and caffeic acids, at retention times of 6.90, 16.34 and 18.11 min, at mg/g of 1.12, 3.77 and 0.39, respectively, with chlorogenic acid appearing most frequently (see Supplementary Fig. S1).

### Effect of roselle extract on bioactive compounds

The results showed that total phenolic, flavonoid, carotenoid, and anthocyanin contents expressed as a value of 11.51 ± 0.49 mg gallic acid equivalent/g extract, 2.93 ± 0.34 mg quercetin equivalent/g extract, 2.38 ± 0.07 mg β-carotene/g extract and 20.82 ± 0.70 mg C3G/g extract, respectively. Bioactive anthocyanin was most frequently found in roselle extract.

### In vitro effect of roselle extract on enzymatic inhibitory activities

Pancreatic lipase inhibitory activities of roselle extract were exhibited in a concentration-dependent manner, with an IC50 value of 2.7 μg/mL. As a comparison, the IC50 of Orlistat was 0.62 μg/mL. Cholesterol esterase inhibitory activities also followed a dose-dependent manner, with an IC50 value of 60.57 mg/mL of roselle extract concentration compared to 0.23 mg/mL for the standard drug simvastatin (see Supplementary Fig. S2).

### Effect of roselle extract on body weight and food intake

As shown in Table 1, final body weight of rats significantly increased in the HFD and HFD + SIM groups compared to the control group, while treatment with roselle extract at doses of 250 and 500 mg/kg BW showed significantly reduced final body weights compared to the HFD and HFD + SIM groups. Food consumption and calorie intake significantly increased in both the high fat diet and simvastatin groups compared to the control group. The roselle extract treated group showed significantly decreased food consumption and calorie intake in a dose-dependent manner compared with the HFD and the HFD + SIM groups. Body weight of rats fed a high fat diet significantly increased compared with the control group, while roselle extract treatment at a dose of 500 mg/kg BW showed significantly decreased gain in body weight. The high fat diet group showed increased Lee index, which is used to evaluate the obesity status in rats. Treatment with roselle extract at 500 mg/kg BW significantly decreased the Lee index compared with the high fat diet group. Roselle extract showed anti-obesity effect in rats fed a high-fat diet.

**Table 1:** Effect of roselle extract on animal and biochemical parameters.

Parameters	Control	HFD	HFD + SIM	HFD + R250	HFD + R500
Initial body weight (g)	299 ± 9.3	317.2 ± 22.8	299.1 ± 14.6	300.1 ± 28.0	294.4 ± 21.6
Final body weight (g)	415 ± 24.7	483.5 ± 9.7***	478.4 ± 6.8***	436.3 ± 24.1^##,+^	416.7 ± 24.3^###,+++^
Food consumption (g/day)	21.35 ± 1.2	25.97 ± 1.3***	26.10 ± 0.3***	22.25 ± 1.5^###,+++^	21.18 ± 1.4^###,+++^
Calories intake (kCal/day)	64.31 ± 3.8	93.62 ± 3.5***	92.12 ± 1.0***	78.5 ± 5.3***^,###,+++^	74.76 ± 4.8***^,###,+++^
Body weight gain (g/day)	4.83 ± 0.5	5.89 ± 0.2**	5.15 ± 0.7	5.13 ± 0.5	4.78 ± 0.5^##^
%Body weight change	179.7 ± 12.9	217.9 ± 4.7**	217.9 ± 27.3*	192.5 ± 20.5	180.0 ± 21.7^##,+^
Lee index	322.6 ± 4.0	334.5 ± 6.2 *	330.8 ± 1.8	328.8 ± 6.7	323.3 ± 8.2^#^
%Liver index	2.85 ± 0.1	4.97 ± 0.4***	4.40 ± 1.0***	4.41 ± 0.1***	3.84 ± 0.2^##^
Serum AST (U/L)	94 ± 12.1	194 ± 42.4***	161.7 ± 33.2**	133.6 ± 12.2^##^	128.4 ± 22.4^#^
Serum ALT (U/L)	44.9 ± 8.1	103.1 ± 16.4***	68.4 ± 30.0^#^	67.8 ± 18.5^#^	64.0 ± 16.4^#^
AST/ALT ratio	2.14 ± 0.3	3.64 ± 1.2*	3.60 ± 0.8*	2.01 ± 0.9^#,+^	1.73 ± 0.7^##,++^
Hepatic TNF-α (ng/mg prot.)	2.82 ± 0.8	7.37 ± 3.0**	1.22 ± 0.1^###^	3.97 ± 1.5^#^	3.56 ± 1.3^##^
Hepatic IL-6 (ng/mg prot.)	3.18 ± 1.7	12.93 ± 5.5***	2.34 ± 1.2^###^	6.81 ± 2.2^##^	6.02 ± 1.8^##^

The data are presented as the mean ± SD values (n = 5–7 per group). The statistical differences as indicated by one-way ANOVA followed by Tukey’s post hoc test. The mean differences were significant compared with the control group (*P < 0.05, **P < 0.01, ***P < 0.001), HFD group (^#^*P* < 0.05, ^##^*P* < 0.01, ^###^*P* < 0.001) and HFD + SIM group (^+^*P* < 0.05, ^++^*P* < 0.01, ^+++^*P* < 0.001).

### Roselle extract alleviated HFD-induced liver histological changes and hepatic lipid accumulations

**Figure 2:**
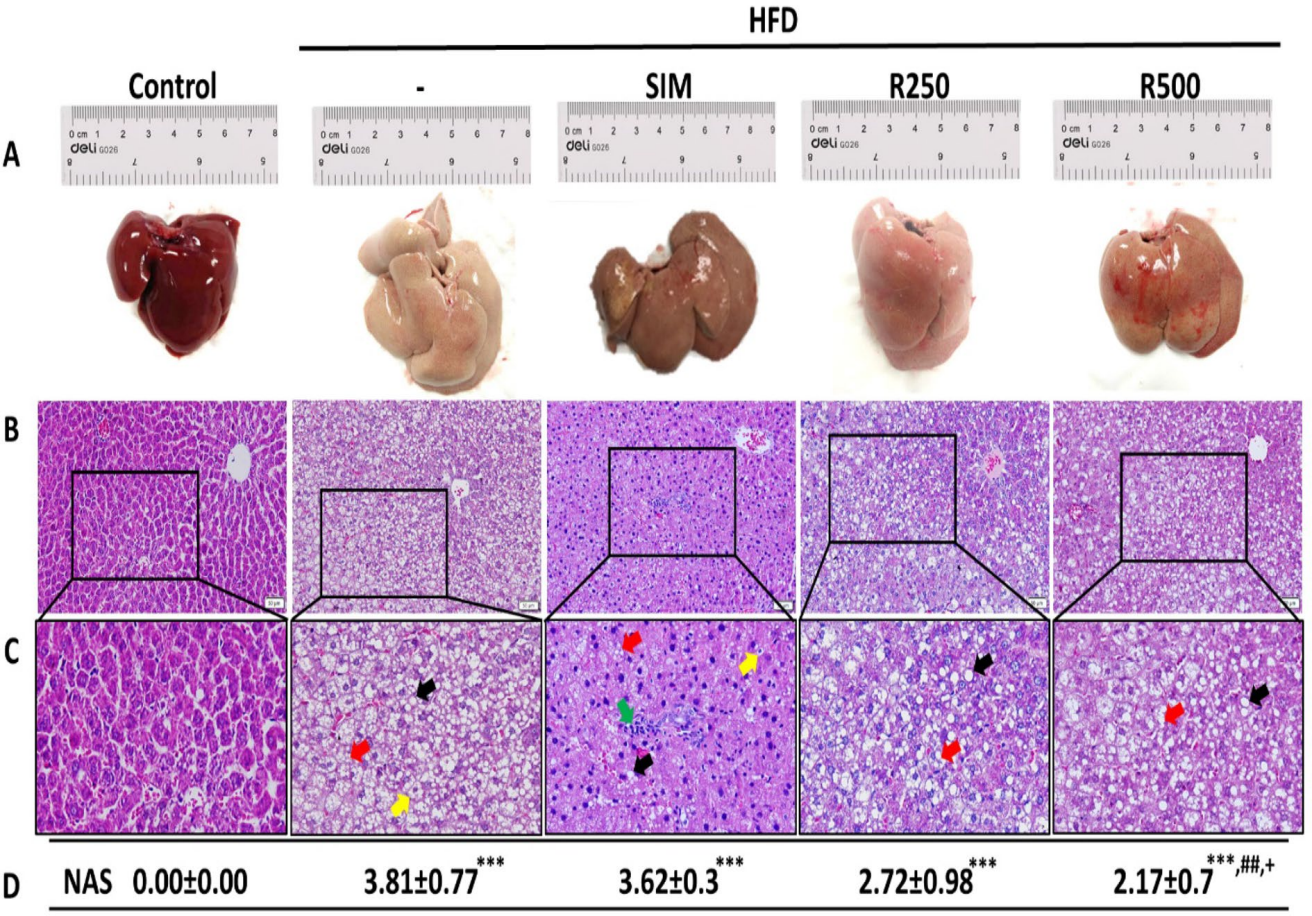
Effect of roselle extract on hepatic steatosis in rats. Rats were fed with normal diet (control), high fat diet (HFD), high fat diet + simvastatin (HFD + SIM), high fat diet + 250 mg/kg BW of roselle extract (HFD + R250), and high fat diet + 500 mg/kg BW of roselle extract (HFD + R500). (**A**) Liver specimens from rats in each group are shown. (**B**) Liver sections with hematoxylin and eosin (H&E) staining (× 200). Scale bars = 50 μm. (**C**) Enlargement areas of liver sections with H&E staining; micro-vesicular steatosis (red arrow), macro-vesicular steatosis (black arrow), lobular inflammation (green arrow), and hepatic ballooning (yellow arrow). (**D**) Non-alcoholic fatty liver disease activity scores (NAS) were evaluated. The data represent the average NAS ± SD of 5 to 7 individual rats per group. NAS were examined in 5 different areas per slide with 2 slides per rat. Statistical analysis was performed with one-way ANOVA followed by Tukey’s post hoc test. The mean difference was significant compared with the control group (***P < 0.001), HFD group (^##^*P* < 0.01) and the HFD + SIM group (^+^*P* < 0.05).

NAFLD activity scores (NAS) used to evaluate histological changes which were diagnosed as NAFL for < 5 and NASH for ≥ 5. As shown in Fig. 2, H&E staining revealed a large amount of micro and macro-vesicular steatosis in the high fat diet group. A significant increase in NAS was observed in the HFD group compared with the control group by H&E staining, indicating that HFD caused NAFLD in rats in the HFD group. Roselle extract treatment at a dose of 500 mg/kg BW showed significantly reduced NAS compared to the HFD group, while simvastatin treatment showed significantly increased NAS compared to the roselle extract group at a dose of 500 mg/kg BW.

**Figure 3:**
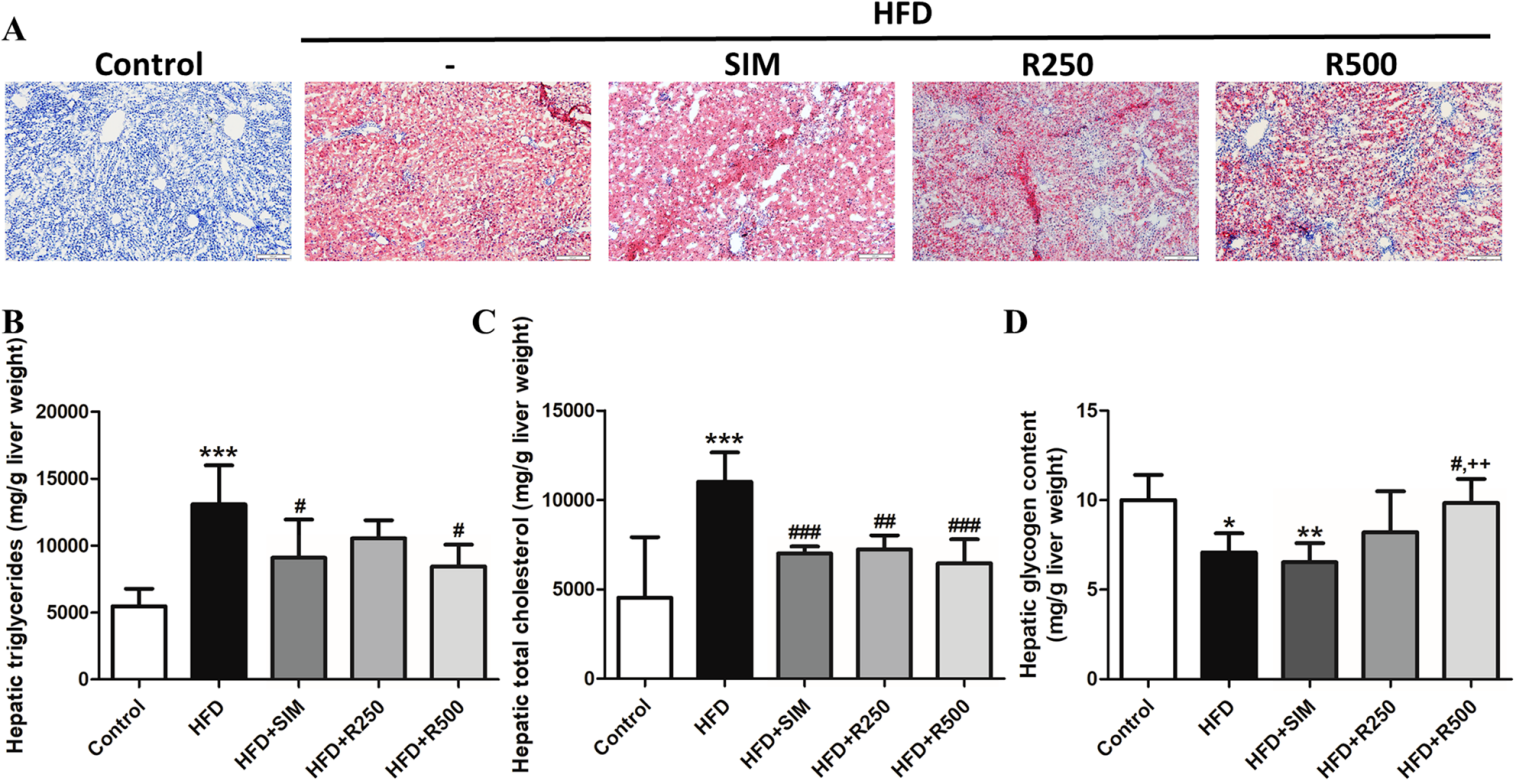
Effect of roselle extract on hepatic lipid accumulation in HFD-induced NAFLD. (**A**) Oil Red O staining (× 100), (**B**) hepatic triglycerides, (**C**) hepatic cholesterol, (**D**) hepatic glycogen content. Lipids and glycogen contents in the liver in rat fed with normal diet (control), high fat diet (HFD), high fat diet + simvastatin (HFD + SIM), high fat diet + 250 mg/kg BW of roselle extract (HFD + R250), and high fat diet + 500 mg/kg BW of roselle extract (HFD + R500). Data were represented as means ± SD from three replications (n = 5–7 per group). Statistical analysis was performed with one-way ANOVA followed by Tukey’s post hoc test. The mean differences were significant compared with the control group (*P < 0.05, **P < 0.01, ***P < 0.001), HFD group (^#^*P* < 0.05, ^##^*P* < 0.01, ^###^*P* < 0.001) and HFD + SIM group (^+^*P* < 0.05, ^++^*P* < 0.01).

Oil Red O is used to stain triglycerides and esterified cholesterols. Results showed that lipid accumulation in the liver increased in the HFD group (Fig. 3A). As shown in Fig. 3B, triglyceride levels increased when rats were fed with a high fat diet. In the presence of different roselle concentrations of 250 and 500 mg/kg BW, triglyceride levels reduced by 19.44% and 35.50%, respectively for the high fat diet group in a dose-dependent manner. Triglyceride levels of the HFD + R500 and HFD + SIM groups significantly decreased compared with the high fat diet group. A high fat diet induced accumulation of total cholesterol in rat liver that significantly increased in the HFD group compared to the control group. Following treatment of roselle at doses of 250 and 500 mg/kg BW, levels of total cholesterol significantly decreased, as shown in Fig. 3C. As a consequence, treatment with roselle extract improved histopathological changes and reduced lipid accumulation in rat livers.

### Effect of roselle extract on liver injury indices in HFD-induced NAFLD

As shown in Table 1, serum aspartate aminotransferase (AST) and alanine aminotransferase (ALT) significantly increased in the high fat diet fed group, while serum AST significantly increased with simvastatin treatment compared to the control group. Treatment with roselle extract at doses of 250 and 500 mg/kg BW significantly reduced levels of serum AST, while serum ALT levels significantly decreased after treatment with simvastatin and roselle extract at doses of 250 and 500 mg/kg BW in rats fed with high fat diet. The AST:ALT ratio significantly increased in the HFD and HFD + SIM groups, while treatment with roselle extract significantly decreased the ratio in a dose-dependent manner. It showed that roselle extracts reduced the severity of liver injury.

### Effect of roselle extract on hepatic glycogen content

A high fat diet significantly reduced hepatic glycogen content compared to the control group, while simvastatin treatment displayed a likelihood of a high fat diet group. Treatment with roselle extract, especially at a dose of 500 mg/kg BW significantly raised levels of hepatic glycogen in rats fed a high fat diet compared to the HFD and HFD + SIM groups, as shown in Fig. 3D.

### Roselle extract improved hepatic antioxidant properties and ameliorated hepatic oxidative stress marker

**Figure 4:**
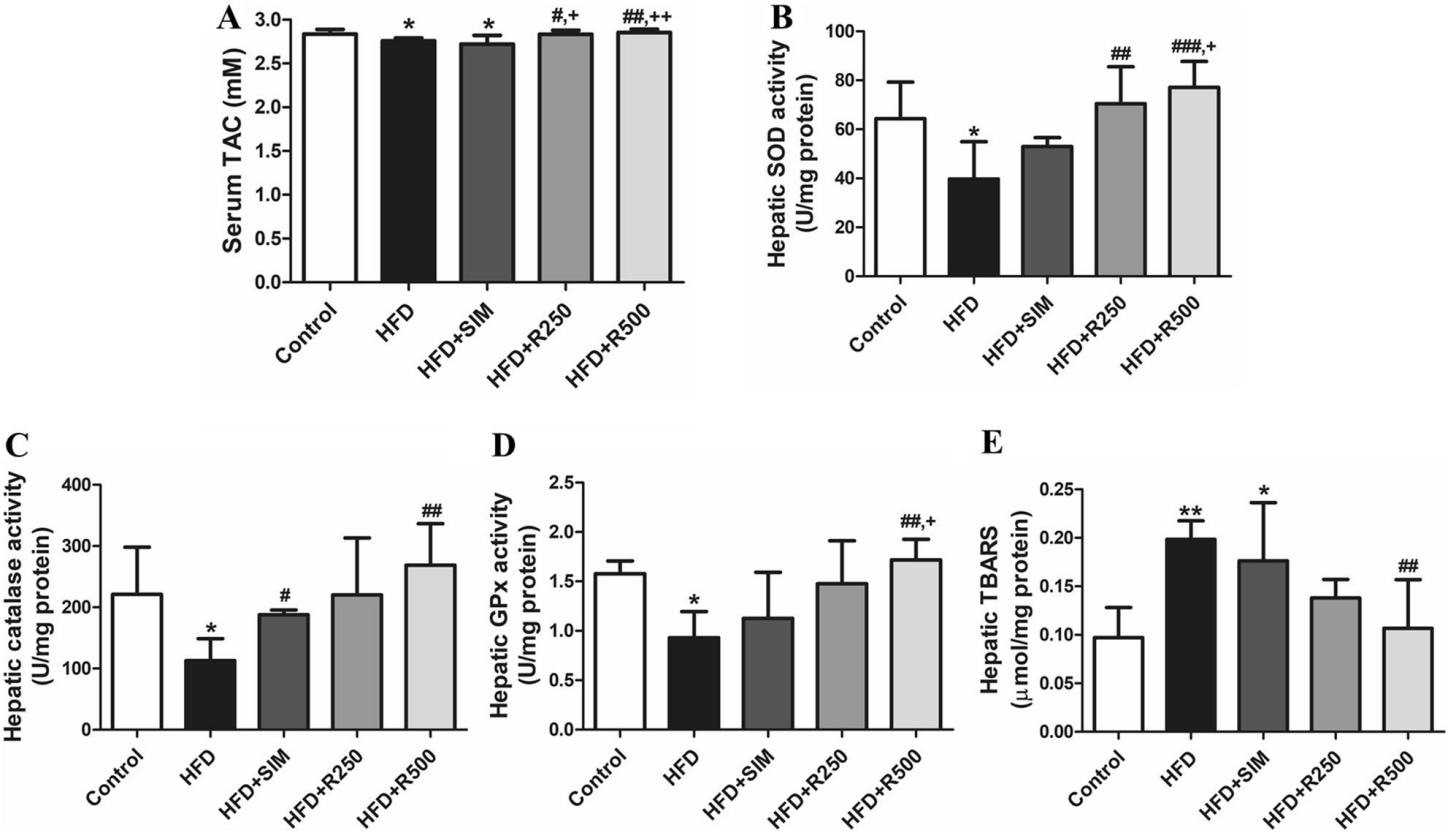
Roselle extract improves antioxidant enzymes and ameliorates lipid peroxidation in liver sample in rat fed high fat diet. (**A**) serum TAC, (**B**) hepatic SOD, (**C**) hepatic catalase, (**D**) hepatic GPx, and (**E**) hepatic TBARS. Data were represented as means ± SD from three replications (n = 5 to 7 per group). Statistical analysis was performed with one-way ANOVA followed by Tukey’s post hoc test. The mean differences were significant compared with the control group (*P < 0.05, **P < 0.01), HFD group (^#^*P* < 0.05, ^##^*P* < 0.01, ^###^*P* < 0.001) and HFD + SIM group (^+^*P* < 0.05, ^++^*P* < 0.01).

As shown in Fig. 4A, serum total antioxidant capacity (TAC) significantly reduced under the high fat diet and simvastatin treatment compared to the control group. Roselle extract treatment at doses of 250 and 500 mg/kg BW significantly increased serum TAC in rats fed with a high fat diet compared to the HFD group.

Hepatic activities of superoxide dismutase (SOD), catalase and glutathione peroxidase (GPx) significantly decreased in rats fed a high fat diet, whereas treatment with roselle extract significantly increased SOD activity in a dose-dependent manner compared to the HFD and HFD + SIM groups. The HFD + SIM and HFD + R500 groups showed significantly increased levels of catalase compared to the HFD group (Fig. 4B–D). GPx activity significantly increased after treatment with roselle extract compared to the HFD and HFD + SIM groups. In summary, roselle extracts increased antioxidant enzymes present in liver tissue.

A high fat diet stimulated liver lipid peroxidation, which significantly increased in the HFD and HFD + SIM groups compared to the control group. Interestingly, treatment with roselle extract at 500 mg/kg BW significantly reduced lipid peroxide levels in rat liver compared with the high fat diet group (Fig. 4E). As a result, lipid peroxidation was alleviated by roselle extract.

### Roselle extract protected against HFD-induced hepatic pro-inflammatory cytokines

A high fat diet significantly increased production of interleukin-6 (IL-6) and tumor-necrosis factor-α (TNF-α) compared to the control group. Pro-inflammatory cytokine levels significantly reduced after treatment of roselle extract at doses of 250 and 500 mg/kg BW and simvastatin compared to the HFD group (Table 1). As a result, roselle extract reduced pro-inflammatory cytokines in rat liver.

### Roselle extract prevented HFD-induced hepatic steatosis, oxidative stress and insulin resistance

**Figure 5:**
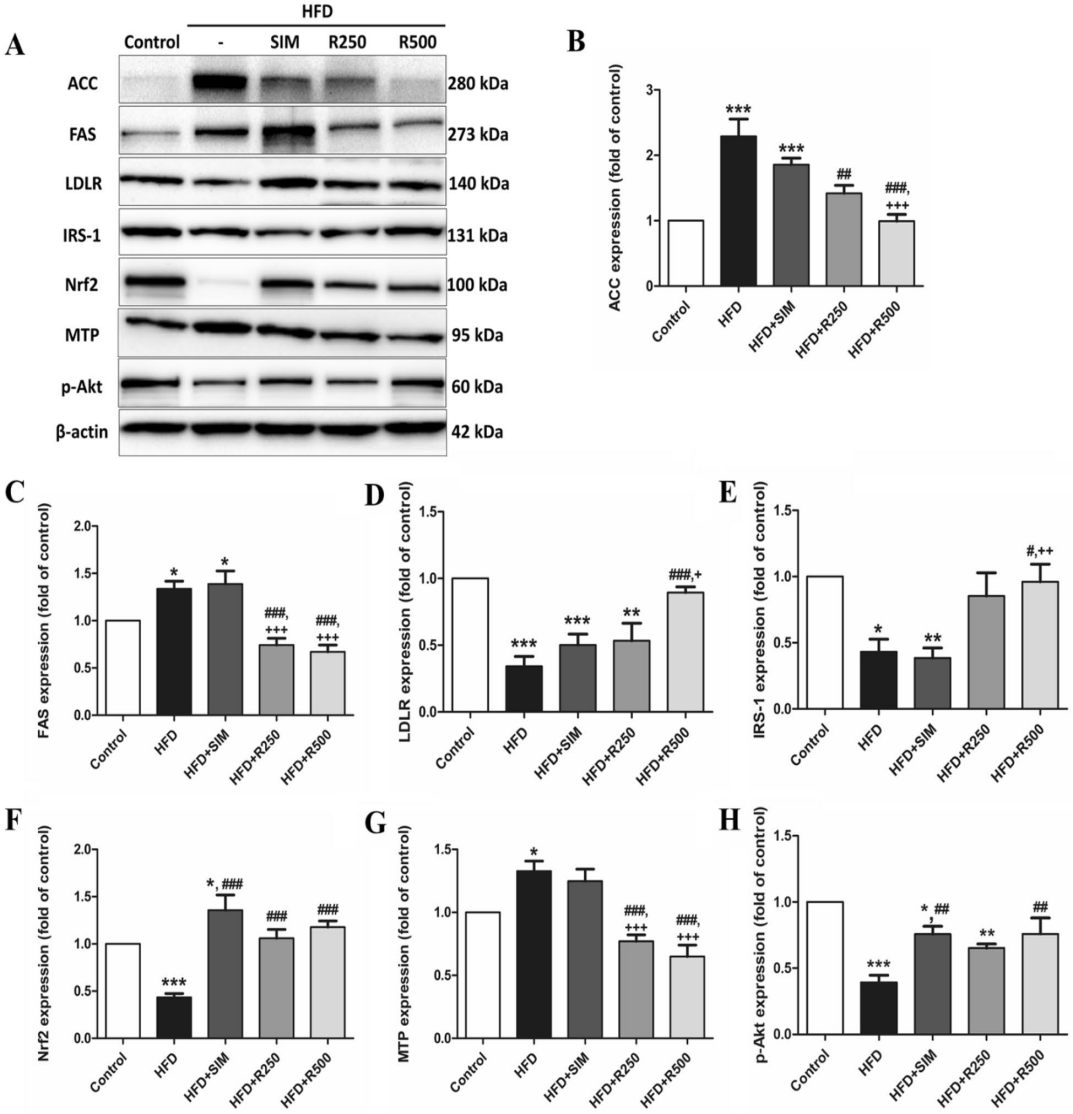
Effect of roselle extract on hepatic protein expression in rats fed high fat diet. (**A**) Band intensity of protein of interest, (**B**) ACC, (**C**) FAS, (**D**) LDLR, (**E**) IRS-1, (**F**) Nrf2, (**G**) MTP, and (**H**) p-Akt (Ser473). The bands are cropped from the original blots which are presented in Supplementary Fig. S3. The data are presented as means ± SD of individual experiments from 5–7 animals per group. The statistical differences as indicated by one-way ANOVA followed by Tukey’s post hoc test. The mean differences were significant compared with the control group (*P < 0.05, **P < 0.01, ***P < 0.001), HFD group (^#^*P* < 0.05, ^##^*P* < 0.01, ^###^*P* < 0.001) and HFD + SIM group (^+^*P* < 0.05, ^++^*P* < 0.01, ^+++^*P* < 0.001).

Expression of protein of interest was represented as band intensity in Fig. 5A. ACC and FAS expression as de novo lipogenesis proteins significantly increased in rats in the HFD and HFD + SIM groups compared to the control group (Fig. 5B,C). Roselle extract treatment significantly decreased the expression of de novo lipogenesis proteins in a dose-dependent manner compared to the HFD group. ACC and FAS expressions in the roselle extract groups were significantly lower compared to the HFD + SIM group. The results demonstrated that roselle extract reduced de novo lipogenesis protein, causing lipid synthesis reduction.

Expressions of cholesterol uptake and transfer proteins including LDLR and MTP are shown in Fig. 5D,G. LDLR expression in the HFD, HFD + SIM and HFD + R250 groups significantly decreased compared to the control group. The roselle extract treatment group at a dose of 500 mg/kg BW significantly increased the expression of LDLR compared to the HFD and simvastatin treated groups. A high fat diet induced MTP expression in the liver that significantly increased in the high fat diet and HFD + SIM groups, and was alleviated by treatment of roselle extract in a dose-dependent manner.

As shown in Fig. 5E, the expression of IRS-1 significantly decreased in the HFD group and the simvastatin treatment group compared with the control group, while IRS-1 expression significantly increased in the 500 mg/kg BW roselle extract treatment group compared to the HFD and simvastatin groups. As shown in Fig. 5H, the expression of p-Akt significantly reduced in the HFD, HFD + SIM and HFD + R250 groups compared to the control group, while treatment with roselle extract, particularly at a dose of 500 mg/kg BW, significantly raised p-Akt expression. The expression of Nrf2 significantly decreased in the HFD group compared to the control group. Treatment with roselle extract and simvastatin significantly increased the Nrf2 expression (Fig. 5F) (see Supplementary Fig. S3). As a result, roselle extract improved insulin resistance and antioxidant defense mechanism in liver tissues.

## Discussion

After high-fat meal consumption, triglycerides are hydrolyzed to free fatty acids and glycerol by lipases, while cholesterol esters are digested by cholesterol esterase to get free cholesterol and transported to the circulation. Previous studies have shown that pancreatic lipase and cholesterol esterase activities increase in rats fed with high fat diet^20,21^. Recent study has shown that the high content of polyphenolic compounds promotes lipase inhibitory activity, which is able to prevent dyslipidemia in hypercholesterolemic mice^22^. In this study, we found that roselle extract had an inhibiting effect on pancreatic lipase and cholesterol esterase enzyme activities, which indicates a possible mechanism of lipid lowering action to alleviate hyperlipidemia. The consumption of a high fat diet may result in the development of dyslipidemia, oxidative stress, insulin resistance and inflammation in the liver. Hepatic steatosis is characterized by lipid droplet accumulation in the hepatocytes. We found that rats fed on a high fat diet for 8 weeks showed a significant increase in hepatic triglyceride and total cholesterol, but they reduce significantly after treatment with roselle extract in a dose dependent manner. Previous studies have indicated that roselle extract contains phytochemical agents including, alkaloids, L-ascorbic acid, anthocyanin, β-carotene, citric acid, hibiscetin, quercetin, stearic acid and wax^17^. In this study, we found many phenolic compounds such as phenolic acids (gallic, caffeic, and chlorogenic acids) and flavonoids (anthocyanins) are present in roselle extract. Phenolic compounds is interesting due to their wide distribution in plants and their health-promoting activities such as antioxidants, and anti-inflammation^19^. Roselle extract has also been used in traditional medicine and pharmacology, which includes lower plasma lipid levels, which reduces liver damage as a hepatoprotection with an antioxidant function^23^. The extracts of the calyces and leaves of 500 mg/kg/day *H. sabdariffa* showed a significant decrease in serum total cholesterol, LDL, VLDL, triglyceride values along with an increase in serum HDL levels in rats fed with high fat diet^24^. The lowering hepatic steatosis in this study may partially from the roselle extracts decreased plasma lipids caused by suppressed adipocytic differentiation in rats fed with high fat diet as described in an earlier study^25^. However, adipose tissue and liver are central tissues in whole body energy metabolism especially liver, which is producing of lipid and cholesterol and then released to blood circulation. Hyperlipidemia is closely related with hepatic lipid accumulation. De novo lipogenesis is the primary metabolic pathway in the liver, and it involves two important enzymes, ACC and FAS, which are responsible for converting excess nutrients into triglycerides. From the present study, we found that roselle reduced hepatic fat accretion by the inhibition of lipogenic enzymes—ACC and FAS expressions, resulting in the reduction of new lipid synthesis in the liver. Therefore, roselle supplementation helps to prevent hepatic steatosis which is induced by a high fat diet.

Consuming a high fat diet induce dyslipidemia and insulin resistance^26^. The mechanism of action of MTP is a catalyst for the critical step in hepatic VLDL synthesis which is secreted into the blood circulation, causing hypertriglyceridemia. In the present study, a high fat diet stimulated MTP expression in the livers of the HFD group while roselle extract inhibited MTP expression in the liver of high fat diet fed rats. Previous studies showed that MTP mRNA increases in the livers of hamsters by long-term high fat diet consumption^27^ and the MTP inhibitors improve dyslipidemia and insulin sensitivity in Zucker fatty rats^26^. Roselle extract may provide as MTP inhibitors, to inhibit the production of lipoproteins, which have been considered important to the treatment of dyslipidemia. LDLR plays a major role in the uptake of LDL, VLDL, and chylomicron remnants which maintains lipid levels in the bloodstream^8^. Previous studies showed that high fat diet reduced LDLR expression in the liver causes hypercholesterolemia^28^. This study found that roselle extract enhances LDLR expression and it shows a greater effect than the simvastatin treatment group. Hence, these benefits represent a novel study of the mechanism of action of roselle extract protect against hyperlipidemia in high fat diet fed rats. In addition to its anti-hypertriglyceridemic and anti-hypercholesterolemic effects, roselle extract also reduced the hepatic fat accumulation of NAFLD rats.

Insulin resistance is defined as insulin receptor damage and the disruption of insulin action, where insulin unable to take up the glucose into the cell, resulting in a hyperglycemia. A high fat diet induced insulin resistance, by reducing the expression of IRS-1, which is an important mediator in insulin signal pathway. When glucose is taken up into the hepatocytes, it is used to provide energy and is stored in the form of glycogen. Previous studies showed that the hepatic glycogen content in the high fat diet fed mice was significantly decreased when compared to a low fat diet group^29^. Our study found that HFD and HFD + SIM decreased the content of liver glycogen in rats. While treatment with roselle extract showed an increase in liver glycogen through activating IRS-1 signal, which increased in the roselle extract treatment group. Insulin binds to insulin receptors on liver cells and activates IRS in the liver. The active phosphoinositide 3-kinase (PI3K) then produces the second messengers phosphatidylinositol 4,5-bisphosphate (PIP2) and phosphatidylinositol-3,4,5-triphosphate (PIP3), which enhance Akt phosphorylation^30^. As a result, the insulin-PI3K/Akt signal pathway plays a critical role in regulating glucose and lipid metabolism in the liver. The activation of IRS-1/Akt signal pathway caused enhanced insulin action and then improved insulin resistance in rats fed with a high fat diet as shown in our study.

High fat diet induces oxidative stress which is the state of imbalance between free radicals and antioxidants in the cell^31^. Overconsumption of fat-rich diet stimulates the β-oxidation of FFAs in the liver, which is associated with the electron transfer in electron transport system to produce the ATP. The intermediates obtained from this pathway may interact with oxygen to produce the accumulation of ROS including superoxide anion radicals and hydrogen peroxide (H_2_O_2_) causing cellular damage. In general, this process is neutralized by the action of antioxidants. Enzymatic (SOD, catalase, GPx, and peroxiredoxin) and nonenzymatic (GSH, thioredoxin, vitamins E and C, flavonoids, and polyphenols) antioxidant systems were found to be effective against ROS^32^. SOD can catalyze superoxide anion to H_2_O_2_ and further hydrolyzed by catalase to water. The GPx enzyme is important in eliminating not only H_2_O_2_, but also lipid peroxides, as well as scavenging free radicals. Nrf2 is an important regulator to work against oxidative stress and promote antioxidant response. The results shown in the present study demonstrated that the roselle extract promotes antioxidant defense via Nrf2 signal pathway against oxidative stress, which decreases the levels of oxidant production and raises the levels of antioxidant enzymes including SOD, catalase, and GPx in the liver in rats fed a high fat diet. The TAC did not show in the HFD + SIM group while roselle extract promotes serum TAC. The active compounds of roselle extract including anthocyanin, phenolic, flavonoid, and carotenoid, show a preventive effect of oxidative damage^33^. These results indicate that roselle extract maintained homeostasis between oxidants and antioxidants in NAFLD rats by increasing the antioxidant enzyme defense mechanism and decreasing lipid peroxidation. Oxidative damage in the liver through the consumption of a high fat diet, leads to liver injury, as evidenced by increased plasma levels of AST and ALT. The presence of these enzymes was measured for assessment of liver abnormal functioning or liver injury. As shown in the present study, levels of the serum AST increased in the HFD and simvastatin group while the enzyme was reduced by the treatment with roselle extract in a dose-dependent manner. High fat diet induced the release of serum ALT while treatment with roselle extract and simvastatin decreased serum ALT. The high fat diet and simvastatin induced serum AST/ALT ratio, while treatment with roselle extract ameliorates liver enzyme injury. The results of the effect of simvastatin on liver function parameters suggest that simvastatin should be used with caution as a treatment for patients with liver disease. Previous studies showed that simvastatin is associated with hepatotoxicity and liver injury after long-term use^34^. Interestingly, this study represents the protective effect of roselle extract on liver injury which induced by a high fat diet, comparing to statin. These results suggest that roselle extract inhibits oxidative damage in the livers of high fat diet fed rats. In addition, HFD-induced ROS is closely related to the pro-inflammatory process that can activate NF-κB signal pathway, and thus induce the production of pro-inflammatory molecules. Previous studies indicated that high fat diet induced pro-inflammatory cytokines such as TNF-α and IL-6, and these cytokines activated the Kupffer cells (liver macrophage)^35^, which causes liver inflammation^36^. In this study, the results showed that the HFD group increased IL-6 and TNF-α, while treatment with roselle extract and simvastatin reduce pro-inflammatory cytokines. These results suggest that roselle extract alleviates the release of pro-inflammatory cytokines and inhibits the progression of NAFLD.

Due to the general factors that may arise owing to sex and age differences, we used male rats at the age of 6 weeks in this study. Male rats are often used in the investigation of metabolic disorders because they display metabolic progression better and faster than female rats^37^. Furthermore, female rats have been shown to undergo hormonal variations, including hormones related with the female reproductive cycle. Such hormones and other variables that affect body composition and/or energy homeostasis, resulting in metabolic differences between males and females^38^. The rats were 6-weeks old when started feeding a high-fat diet and administering the extract. Previous research has shown that diet-induced obesity can be induced in male Sprague–Dawley rats aged 5–6 weeks^39–42^. It is shown that female and male rats reach sexual maturity at week 5–6, respectively^43^. Lee’s study showed that male Sprague–Dawley rats weighing 250–270 g, approximately 5–6 weeks of age, were induced to NAFLD at 8 weeks^44^. Several studies have shown that the metabolic parameters including hepatic enzyme activities, serum and hepatic lipid profiles, glucose, insulin, glucagon and adiponectin levels were not altered in the different age groups of rats fed with a high fat diet^45–48^. It can be seen that there is no difference in metabolic parameters between young and adult rats with high fat diet administration.

Previous studies showed that 450 g of roselle extract was administered 3 times per day over 4 weeks^49^, and 500 g of fresh calyxes were administered once daily as a treatment for hypercholesterolemic subjects^50^. The duration times of these previous studies ranged from 15 to 90 days for the administration roselle extract^51,52^. Our study suggests that a daily administration of 500 mg/kg BW of roselle extract for 8 weeks, which is equivalent to 4.8 g of roselle extract per day is recommended for a 60 kg human. This dosage prevents NAFLD, insulin resistance, inflammation, and oxidative stress in rats fed with a high fat diet. The present study found roselle extract strongly exhibited a protective effect against NAFLD and it was found comparable to the standard medicine; simvastatin. Interestingly, the greater benefit of roselle extract we found in the present study was represented in terms of anti-hepatic steatosis, antioxidant properties, and improved insulin resistance than the effect of simvastatin. However, it is recommended for further human trials to approve its preventive efficacy.

**Figure 6:**
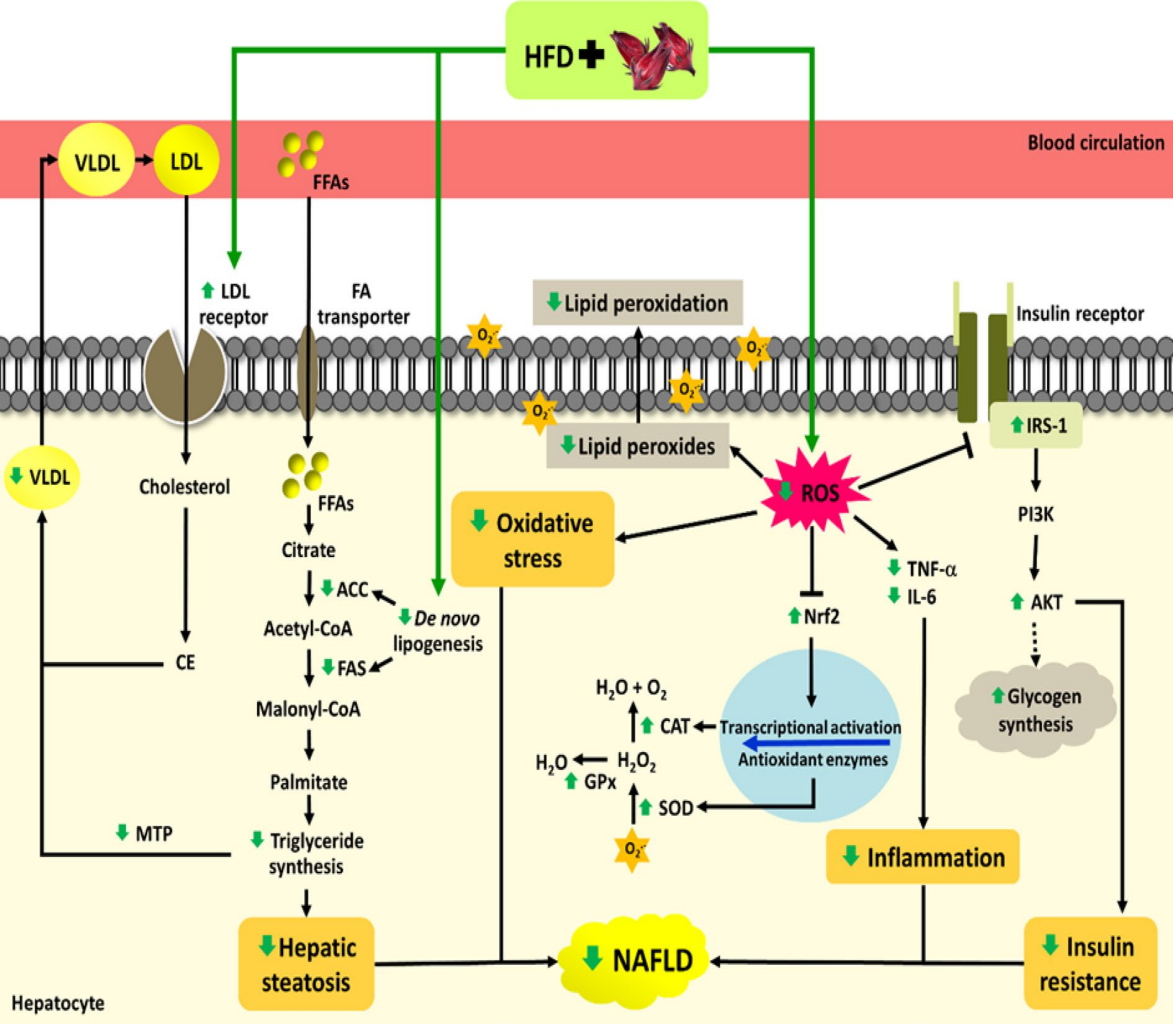
Schematic summary of the mechanism of action of roselle extract on NAFLD rats induced by high fat diet. Roselle extract inhibits hepatic steatosis via inhibiting de novo lipogenesis pathway leads to decreased lipid accumulation in the liver. Roselle extract prevents the increase of ROS and activates antioxidant protein regulators, against oxidative damage and inflammation in rats fed with a high fat diet. Roselle extract improves insulin resistance and promotes antioxidant properties by partial activating IRS-1/Akt and Nrf2 signal pathways.

In summary as shown in Fig. 6, our findings demonstrated that roselle extract has the potential to protect against NAFLD in rats fed a high fat diet by alleviating lipid accumulation through the inhibition of the de novo lipogenesis proteins, reducing lipid peroxidation, inflammation, triglyceride transfer protein and rising antioxidant enzymes, enhancing antioxidant properties, promoting protein related with cholesterol uptake, and improving insulin resistance in rats fed a high fat diet.

## Methods

### Roselle extract preparation

The roselle extract powder was purchased from Specialty, a natural products company, Chonburi, Thailand. The 250 mg of roselle extract powder was dissolved in 2 mL of distilled water which was administered daily to the rats.

### HPLC analysis

Roselle extracts were determined by reverse-phase high performance liquid chromatography which was carried out using HPLC with diode array detector on a Hypersil ODS column (250 × 4.0 mm i.d.; 5 µm particle size). A gradient elution was carried out with solvent A (methanol) and solvent B (1% acetic acid in deionized water) for the analysis of gallic, caffeic and chlorogenic acids. The flow rate was 1.0 mL/min, and the absorbance was measured at a wavelength of 280 nm. The amount of gallic, caffeic and chlorogenic acids were determined by comparing the peak area of the extract solution with the peak area from the standard curve.

### Determination of bioactive compounds in roselle extract (in vitro)

#### Determination of total phenolic content

The total phenolic content was determined using Folin–Ciocalteu assay as described earlier^53^. The roselle extract solution was added into a 96-well plate, followed by 10% Folin–Ciocalteu reagent and 7% sodium carbonate. The solutions were mixed together and kept in the dark for 30 min, then the absorbance was measured at 760 nm using a microplate reader (Perkin Elmer Inc., USA). The total phenolic contents were expressed as mg gallic acid equivalents per gram extract (GAE).

#### Determination of total flavonoid content

The total flavonoid content was determined using the aluminum chloride colorimetric method. The roselle extract solution was added to a 96-well plate, then mixed with aluminum chloride, 10% sodium acetate and distilled water. The solutions were incubated and protected from light for 15 min, then the absorbance was measured at 430 nm. The total flavonoid contents were expressed as milligram quercetin equivalents per gram extract (QE).

#### Determination of total carotenoid content

The total carotenoid content was also determined by adding the roselle extract solution (pretreatment by extraction with ether and hexane) into a 96-well plate, then the absorbance was measured at 450 nm using a microplate reader. The total carotenoids content was expressed as milligram β-carotene equivalents per gram of extract (mg β-carotene/g extract).

#### Determination of total anthocyanins content

The total anthocyanins content in the extract was determined using the pH differential method as described and modified from Lee et al.^54^. The roselle extract solution was diluted with 0.025 M potassium chloride solution at pH1, and in the same manner, the extract was diluted with 0.4 M sodium acetate at pH4.5. The absorbance was then measured at 535 and 700 nm using a microplate reader. The total anthocyanin content was expressed as milligram cyanidin-3-glucoside equivalents per gram of extract (mg C3G/g extract).

### Pancreatic lipase inhibition assay (in vitro)

The pancreatic lipase inhibition assay procedure was described earlier^55^. Various concentrations of roselle extract and Orlistat solutions were mixed with assay buffer, pancreatic lipase enzyme, and p-nitrophenyl laurate, which was followed by the mixtures being incubated at 37 °C for 2 h. After incubation, the mixtures were centrifuged at 16,000 rpm at 25 °C for 5 min. The supernatants were collected and their absorbances were measured using a microplate reader at 400 nm. Orlistat was used as a positive control. Calculation of %inhibition = ((A − B)/A) × 100 where “A” is an optimal density of blank solution, “B” is the optimal density of the sample solution.

### Cholesterol esterase inhibition assay (in vitro)

Cholesterol esterase inhibition procedure was modified from Gururaja et al.^56^. This assay was performed in triplicate, and simvastatin was used as a positive control. Various concentrations of roselle extract and simvastatin solutions were incubated with reagent mixtures of sodium phosphate buffer pH7.0, 5.16 mM taurocholic acid, and 2 mM p-nitrophenyl butyrate. Then cholesterol esterase was added, to initiate the reaction and was incubated at room temperature for 5 min, and the absorbance was measured at 405 nm. Cholesterol esterase inhibition was compared with a simvastatin standard curve. Calculation of %inhibition = ((A − B)/A) × 100 where “A” is the optimal density of the blank solution, “B” is the optimal density of the sample solution.

### Animals and experimental design

Thirty-five 4-weeks old male Sprague–Dawley rats were ordered from the Nomura Siam International, Bangkok, Thailand. This study followed ARRIVE guidelines, and all animal experiments were approved by the ethics committee from the Naresuan University Center for Animal Research (NUCAR) before the trial began under the NUAE-580713 project tract number. All experiments were carried out in conformity with all applicable regulations and guidelines. The animals were housed under controlled conditions to acclimatize and handling for two weeks, before the experiment began. The rats were 6-weeks old at the beginning of the experiment. The rats were randomly divided into 5 groups: (1) control group (C; n = 7) were fed a normal diet, (2) high-fat diet group (HFD; n = 7) were fed a high-fat diet, (3) high-fat diet treated with simvastatin group (HFD + SIM; n = 7) were fed a high fat diet with 40 mg/kg/day of simvastatin by oral gavage, (4) high-fat diet treated with roselle group (HFD + R250; n = 7) were fed a high fat diet with 250 mg/kg/day of roselle extract by oral gavage, (5) high fat diet were treated with roselle group (HFD + R500; n = 7) were fed a high fat diet with 500 mg/kg/day of roselle extract by oral gavage. The composition of high fat diet was modified from Janson et al*.*^25^. Briefly, the high fat diet contained 20 g of fat/100 g of pellets (4.5 g of oil, 14 g of milk fat and 1.5 g of cholesterol) which 36% of total energy intake provided by fats while the control diet (4.5 g of oil) provide 10% of energy from fats. The animals in the experimental group received a high fat diet with roselle feeding by intragastric gavage on a daily basis. An equal volume of distilled water was given to the control and HF groups through the same route. The food intake was monitored daily, and their body weights were measured every week. After 8 weeks, the rats were fasted for 12 h and then euthanized; all of their blood, as well as their livers, were collected for experimental testing and recorded their body and liver weights. The liver index was calculated as absolute liver weight (g)/body weight (g) × 100%. The rat’s livers were dissected for histological analysis and stored at − 80 °C until required for biochemical analysis. The doses of roselle extract at 250 and 500 mg/kg BW in this study were converted to a human equivalent dose (HED), which suggests that human consumption would be 40 and 80 mg/kg BW, respectively^57^. Therefore, the clinical doses at 2.4 and 4.8 g of roselle extract per day is recommended for a 60 kg human.

### Determination of liver injury indices

After the rats were euthanized, their blood was collected and centrifuged at 3000 rpm for 30 min at 4 °C. Then the supernatant was collected and the serum AST (Aspartate aminotransferase) and ALT (Alanine aminotransferase) were measured by Medilab (Phitsanulok, Thailand).

### Hematoxylin and eosin (H&E) staining

The liver in 10% neutral buffered formalin were embedded in paraffin. Liver samples were sectioned into 5 μm. The result was observed under a light microscope and evaluated as NAFLD activity score (NAS) as described earlier^58^ based on 3 features: hepatic steatosis (0–3), lobular inflammation (0–3), and hepatic ballooning (0–2). In brief, hepatic steatosis is referred to the presence of ≥ 5% of lipid droplets accumulate in the hepatocytes. Lobular inflammation is characterized as an inflammatory cell infiltration and aggregation in the liver. The swollen hepatocytes and rarefied cytoplasm are known as hepatic ballooning. NAS were examined in 5 different areas per slide and then performed 2 slides per rat with 5 to 7 rats per group.

### Oil Red O staining

Oil Red O is an oil-soluble dye used for staining neutral triglycerides and lipids such as esterified cholesterols. Fresh liver samples were frozen to an optimum cutting temperature (OCT), then stained with Oil Red O for lipid analysis. The livers were rapidly harvested from the rats, and then stored in 15% sucrose. The stained liver samples were observed under a light microscope and analyzed by image J.

### Sample preparation for biochemical analysis

Liver samples of 200 mg were homogenized in phosphate buffered saline (PBS) pH 7.0 buffer on ice by using ultrasonicator. Following centrifugation at 22,000 rpm for 20 min at 4 °C, the supernatant was obtained aliquots which were stored at − 80 °C until subjected to biochemical analysis. The measure of the protein in the homogenized liver samples which was then compared to BSA standard by Bradford assay.

### Determination of antioxidant properties and lipid peroxidation

#### Serum total antioxidant capacity

The TAC was determined by ABTS (2,2′-Azino-bis (3-ethylbenzothiazoline-6-sulfonic acid) diammonium salt) assay. ABTS solution was prepared by adding potassium persulphate (K_2_S_2_O_8_) solution and then the reagent was incubated in the dark for 12–16 h. The ABTS working solution was prepared by the dilution of ABTS with distilled water and the absorbance was read at 734 nm. The serum was added into a 96-well plate then the ABTS working solution was added and incubated at room temperature in the dark for 30 min. Serum TAC was compared to the Trolox standard curve.

#### Hepatic superoxide dismutase activity

Total SOD activity in the homogenized liver sample were measured by spectrophotometry at 420 nm. After the addition of Tris buffer in 1 mM EDTA, then ddH_2_O and 50 mM pyrogallol in 10 mM hydrochloric acid (HCl) were added, respectively. The homogenized liver samples were added and then mixed. The absorbance was recorded continuously for two minutes using the method of Li^59^. Analysis of SOD activity (Units/mL) were compared with the SOD standard curve and normalized by individual protein concentration.

#### Hepatic catalase activity

Total catalase activity in the homogenized liver sample were measured by the method described earlier^60^. The mixtures of PBS and 30% hydrogen peroxide (H_2_O_2_) was added to the cuvette, followed by the liver sample. The absorbance was read by spectrophotometry at 240 nm and was recorded continuously for 5 min. Analysis of catalase activity (Units/mL) was compared with the catalase standard curve and normalized by individual protein concentration.

#### Hepatic glutathione peroxidase activity

The GPx activity in the homogenized liver sample were measured by the modified method of Samad et al.^61^. The samples were added with 0.2 M phosphate buffer, 4 mM reduced Glutathione, 10 mM sodium azide, and 2.5 mM hydrogen peroxide then were mixed and incubated at 37 °C for 5 min. After the incubation, trichloroacetic acid was added into the test tubes, following centrifuged at 3000 rpm for 5 min. The supernatant was collected and mixed with 5,5′-Dithiobis (2-nitrobenzoic acid) (DTNB), then the absorbance was read at 412 nm. Analysis of GPx activity (Units/mL) was compared with the GPx standard curve and normalized by individual protein concentration.

#### Hepatic lipid peroxidation

The liver sample was mixed with the solutions containing 2-thiobarbituric acid, HCl and trichloroacetic acid as described earlier^51^. The mixtures were then incubated for 15 min at 95 °C, and then cooled on ice. After centrifugation, the supernatant was collected and measured by microplate reader at 535 nm. The measurement of lipid peroxidation was carried out by comparing with 1,1,3,3-tetramethoxypropane standard and normalized by individual protein concentration.

### Determination of liver inflammation

The homogenized liver samples were determined by the ELISA commercial kit according to the manufacturer's protocols for the determination of interleukin-6 (IL-6) and tumor necrosis factor-α (TNF-α) from Sigma-Aldrich, MO, USA. Liver inflammation levels were normalized by individual protein concentration.

### Hepatic lipids measurement

Lipid extraction procedure was modified from Folch’s method using 1:2 methanol–chloroform (v/v) and was separated into three phases. The lower phase is retained and then dried by nitrogen gas. Dried lipids were dissolved in methanol-chloroform mixture. The commercial kits (Human Gesellschaft fur biochemical und diagnostica mbH, Germany) were used for the measurement of triglycerides, and total cholesterol in liver.

### Glycogen content assay

Glycogen was extracted from fresh liver tissues by modified from Shokri-Afra et al.^62^. The addition of 30% KOH in liver samples were then boiled for 30 min. An ice-cold ethanol solution was then added and was centrifuged at 18,000 rpm for 20 min. The pellets were dissolved in distilled water and phenol reagent. A sulfuric acid solution was added and incubated on ice for 30 min. Glycogen content was determined by glucose assay kit (Sigma-Aldrich, MO, USA).

### Western blot analysis

The liver samples were lysed in RIPA buffer (Sigma-Aldrich, MO, USA). Total protein concentration was determined by BCA protein assay kit. The processed samples were loaded and run on 8% SDS–polyacrylamide gel, and then transferred to PVDF membranes (Merck, MA, USA). The membranes were blocked with 5% skimmed milk. Next, the membranes were incubated at 4 °C overnight with different primary antibodies: ACC (1:500, Cell signal, MA, USA), FAS (1:500, Cell signal, MA, USA), LDLR (1:500, Abcam, Cambridge, UK), IRS-1 (1:500, Elabscience Biotechnology Co., Ltd, TX, USA), Nrf2 (1:500, Affinity Biosciences Ltd, OH, USA), MTP (1:3000, BD Biosciences, NJ, USA), phospho-Akt (Ser473) (1:500, Cell signal, MA, USA), and β-actin (1:2000, Elabscience Biotechnology Co., Ltd, TX, USA). The membranes were washed with TBS-0.1% tween 20 and were incubated with horseradish peroxidase (HRP) conjugated secondary antibodies for 2 h. After this, the membranes were washed with TBS-0.1% tween 20, and covered HRP substrate reagent (Merck, MA, USA). The Images were detected by Gel Doc XR+ and ChemiDoc XRS+ Imaging Systems (Bio-Rad, CA, USA). The band density from the target protein and the housekeeping protein/loading control (β-actin) were quantified for each batch using Image Lab Software 6.0.1 (Bio-Rad). The ratio of the target protein to β-actin is then calculated, yielding the normalized density of the loading control (NDL) and calculated the fold difference by dividing the NDL from each lane by the NDL from the control sample in that batch. This ratio yields the difference in sample load between the reference and other lanes. All experiments were performed in duplicate to represent the mean ± SD of each rat of all data (n = 5–7 animals/group).

### Statistical analysis

The data were analyzed using the GraphPad Prism 5.01. (GraphPad Software, USA). The data are presented as mean ± standard deviation (SD) of 5–7 animals per group (replications as indicated in the figure legend of each experiment). An analysis of variance (One-way ANOVA) was used to detect differences. Tukey’s test was used to correct for multiple comparisons. A value of *P* < 0.05 was considered statistically significant.

## Supplementary Information

Supplementary Figures.
