# Supplementary material for: Hibiscus sabdariffa extract improves hepatic steatosis, partially through IRS-1/Akt and Nrf2 signaling pathways in rats fed a high fat diet
Source: Sci Rep. 2022 Apr 29;12:7022. doi: 10.1038/s41598-022-11027-9 (PMC9054782; doi:10.1038/s41598-022-11027-9)
Supplement: Supplementary file 1 — Supplementary Figures. [file 41598_2022_11027_MOESM1_ESM.pdf]

Supplementary Information

Hibiscus sabdariffa extract improves hepatic steatosis, partially through IRS-1/Akt and Nrf2 signaling pathways in rats fed a high fat diet

Janjira Prasomthong[1], Nanteetip Limpeanchob[2,5], Supawadee Daodee[3], Pennapa Chonpathompikunlert[4], and Sakara Tunsophon[1,5,*]

[1]Department of Physiology, Faculty of Medical Science, Naresuan University, Phitsanulok 65000, Thailand.

[2]Department of Pharmacy Practice, Faculty of Pharmaceutical Sciences, Naresuan University, Phitsanulok 65000, Thailand.

[3]Division of Pharmaceutical Chemistry, Faculty of Pharmaceutical Sciences, Khon Kaen University, Khon Kaen 40000, Thailand.

[4]Expert Center of Innovative Health Food (Innofood), Thailand Institute of Scientific and Technological Research (TISTR), Pathumthani 12120, Thailand.

[5]Center of Excellence for Innovation in Chemistry, Naresuan University, Phitsanulok, 65000, Thailand.

*Corresponding author:
Sakara Tunsophon, PhD
Email: sakarat@nu.ac.th

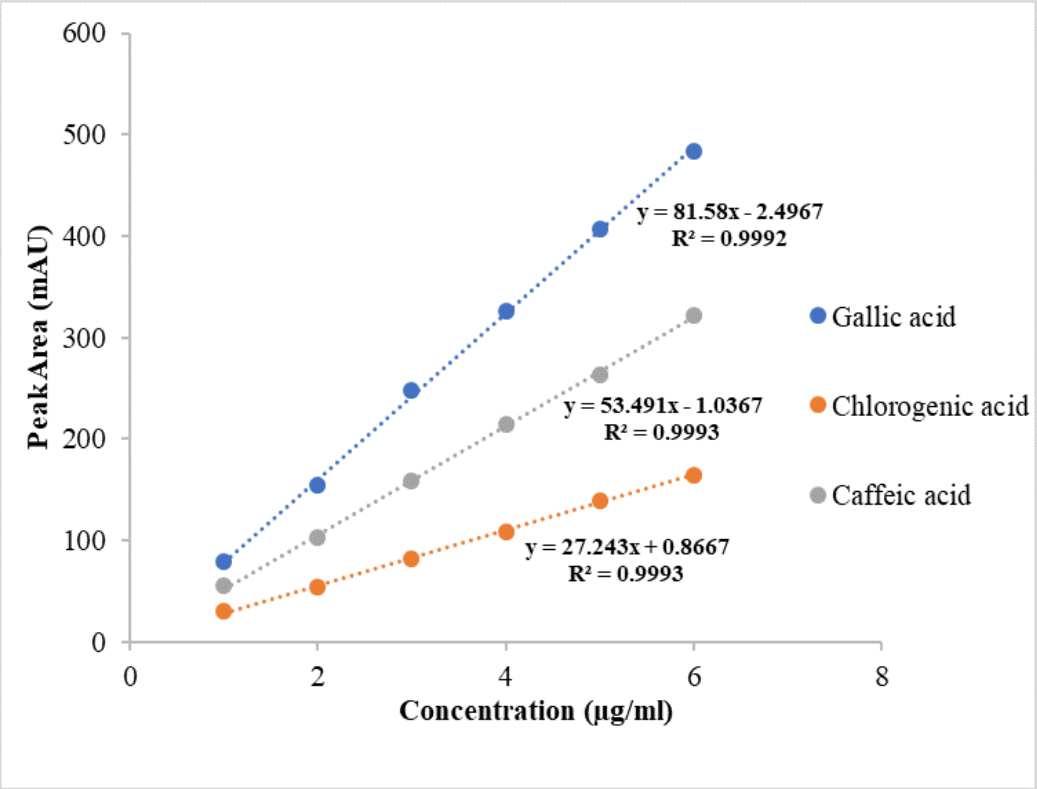

Supplementary Figure S1

Supplementary Figure 1. Standard curve of standard gallic acid, chlorogenic acid, and caffeic acid solutions. Standard curves represent the relationship between HPLC peak area (mAU) and concentrations of gallic, chlorogenic, and caffeic acids (µg/ml).

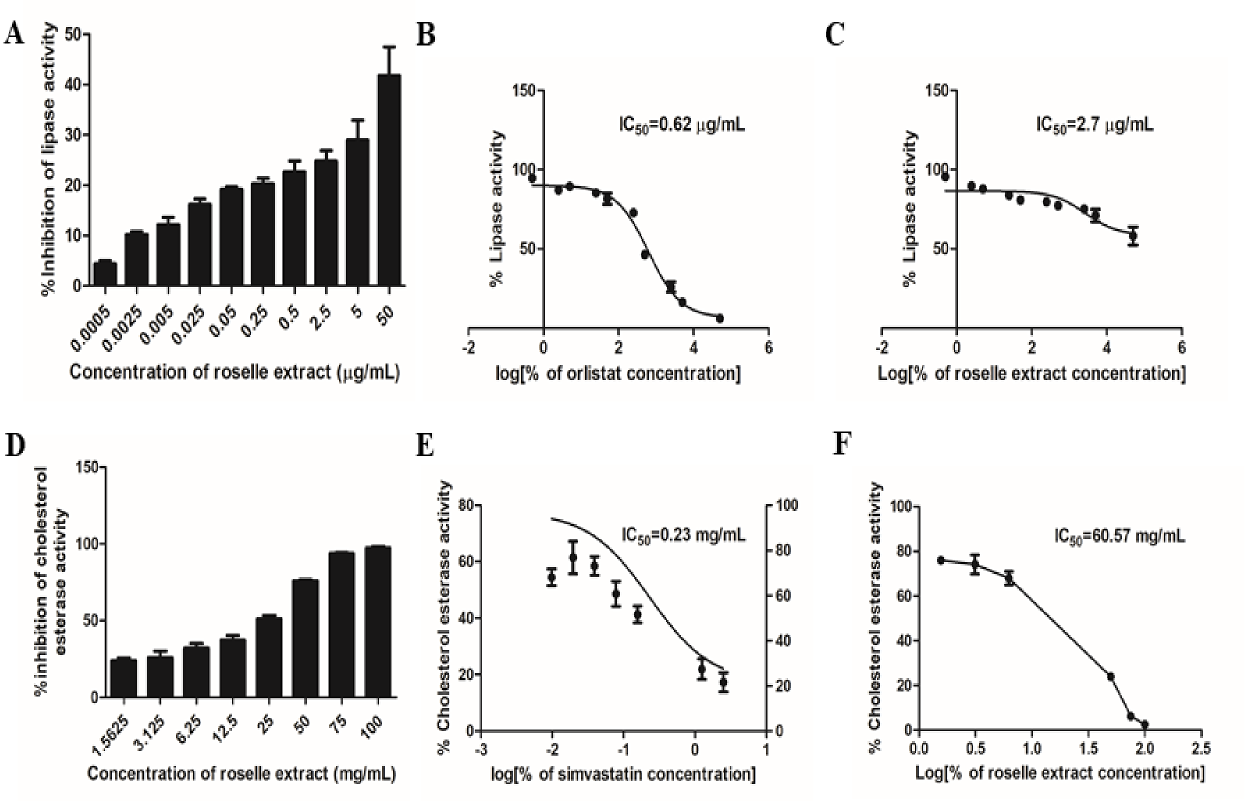

Supplementary Figure S2

Supplementary Figure 2. Effect of roselle extract on enzymatic inhibitory activities *(In vitro)*. Inhibitory effect of roselle extract on lipase and cholesterol esterase activities compared to positive control drugs such as orlistat and simvastatin, respectively. (A) % Lipase activity inhibition by roselle extract, (B) IC50 of orlistat on lipase activity, (C) IC50 of roselle extract on lipase activity, (D) % Cholesterol esterase activity inhibition by roselle extract, (E) IC50 of simvastatin on cholesterol esterase activity, (F) IC50 of roselle extract on cholesterol esterase activity.

Western blot data

Liver proteins were loaded and run on 8% SDS-polyacrylamide gel, and then transferred to PVDF membranes. The membranes were blocked with 5% skimmed milk. Following confirmation of the molecular weights of proteins, the membranes were cropped and incubated at 4 °C overnight with different primary antibodies: ACC, FAS, LDLR, IRS-1, Nrf2, MTP, phospho-Akt (Ser473). The membranes were washed with TBS-0.1% tween 20 and were incubated with horseradish peroxidase (HRP) conjugated secondary antibodies for 2 hours. The membranes were then washed with TBS-0.1% tween 20, and covered HRP substrate reagent. The Images were detected by Gel Doc XR+ and ChemiDoc XRS+ Imaging Systems (Bio-Rad, CA, USA) and band intensities were analyzed using Image Lab Software 6.0.1, Bio-Rad. All measurements were normalized to β-actin. We have shown the blots of repeated data with multiple exposure times in the supplementary figures.

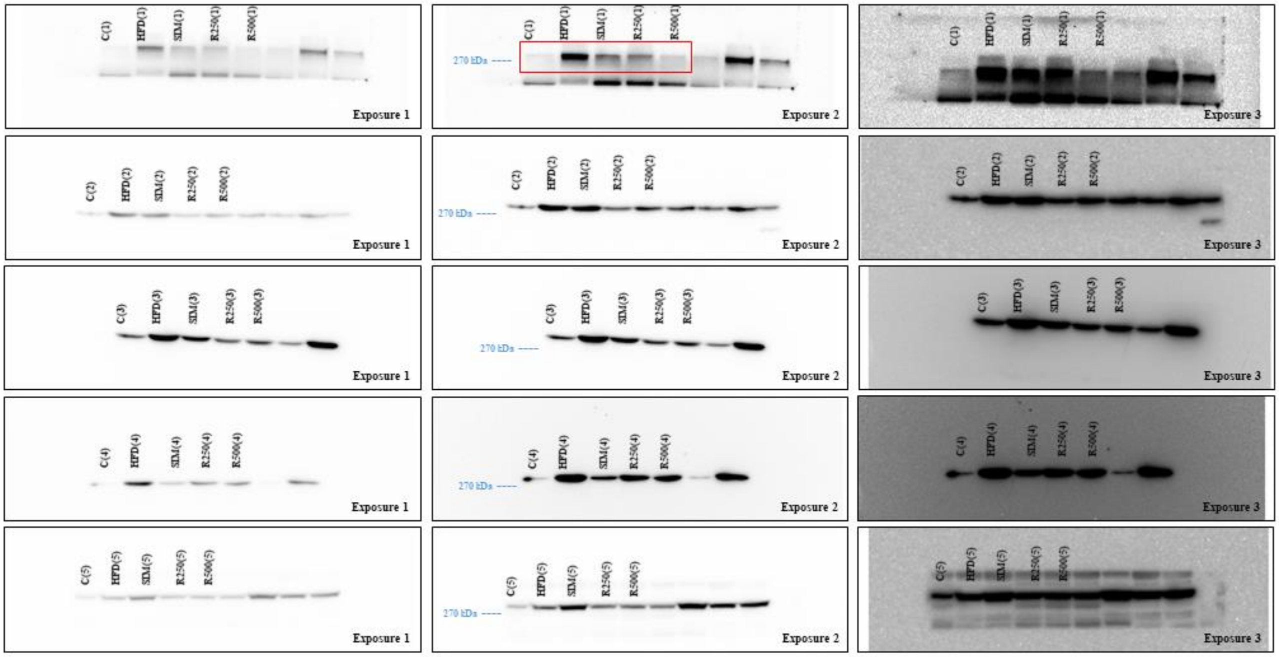

Supplementary Figure S3

Supplementary Figure 3A: Original (uncropped) images of western blotting membranes for Figure 5A (ACC). Cropped blot shown in Figure 5A (ACC) is indicated by the framed region. The blots of repeated data with difference exposure times were shown in the supplementary figure (exposure 1, 2, and 3 at 30, 120, and 300 sec, respectively). The livers of each group were analyzed with antibodies against ACC on Western blot. The data are used to present the average of 5 individual rats ± SD (n=5 per group). The statistical analysis was performed with one-way ANOVA followed by Tukey's *post hoc* test.

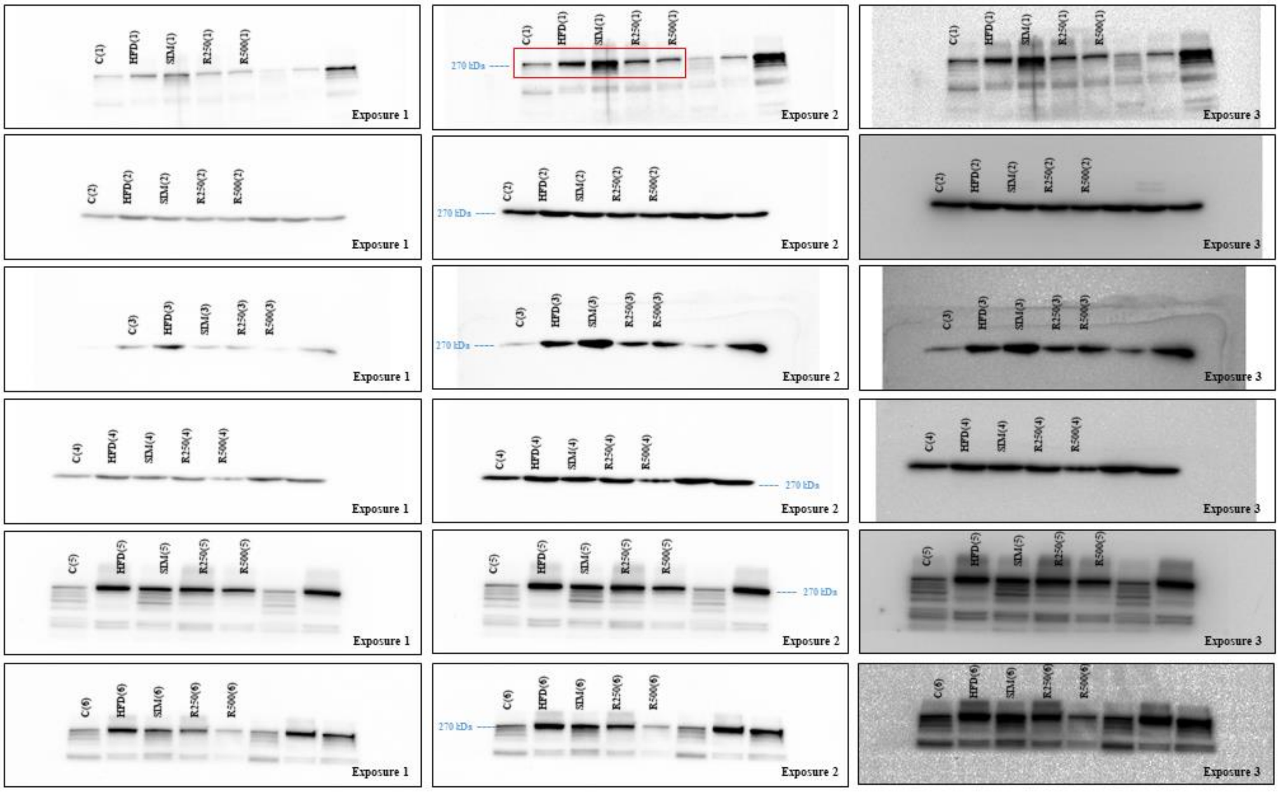

Supplementary Figure 3B: Original (uncropped) images of western blotting membranes for Figure 5A (FAS). Cropped blot shown in Figure 5A (FAS) is indicated by the framed region. The blots of repeated data with difference exposure times were shown in the supplementary figure (exposure 1, 2, and 3 at 30, 120, and 300 sec, respectively). The livers of each group were analyzed with antibodies against FAS on Western blot and normalized with β-actin. The data are used to present the average of 6 individual rats ± SD (n=6 per group). The statistical analysis was performed with one-way ANOVA followed by Tukey's *post hoc* test.

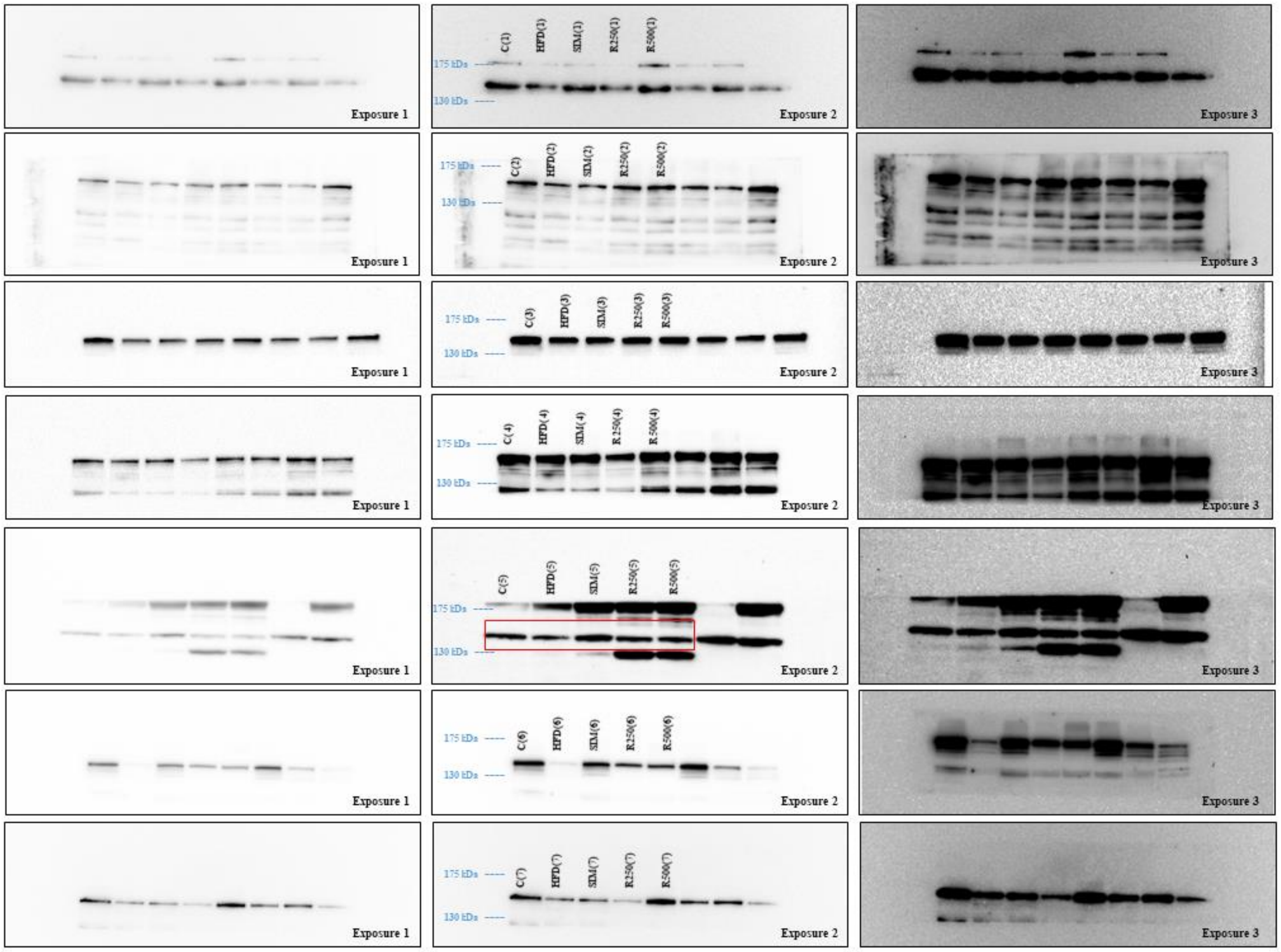

Supplementary Figure 3C: Original (uncropped) images of western blotting membranes for Figure 5A (LDLR). Cropped blot shown in Figure 5A (LDLR) is indicated by the framed region. The blots of repeated data with difference exposure times were shown in the supplementary figure (exposure 1, 2, and 3 at 30, 120, and 300 sec, respectively). The livers of each group were analyzed with antibodies against LDLR on Western blot and normalized with β-actin. The data are used to present the average of 7 individual rats ± SD (n=7 per group). The statistical analysis was performed with one-way ANOVA followed by Tukey's *post hoc* test.

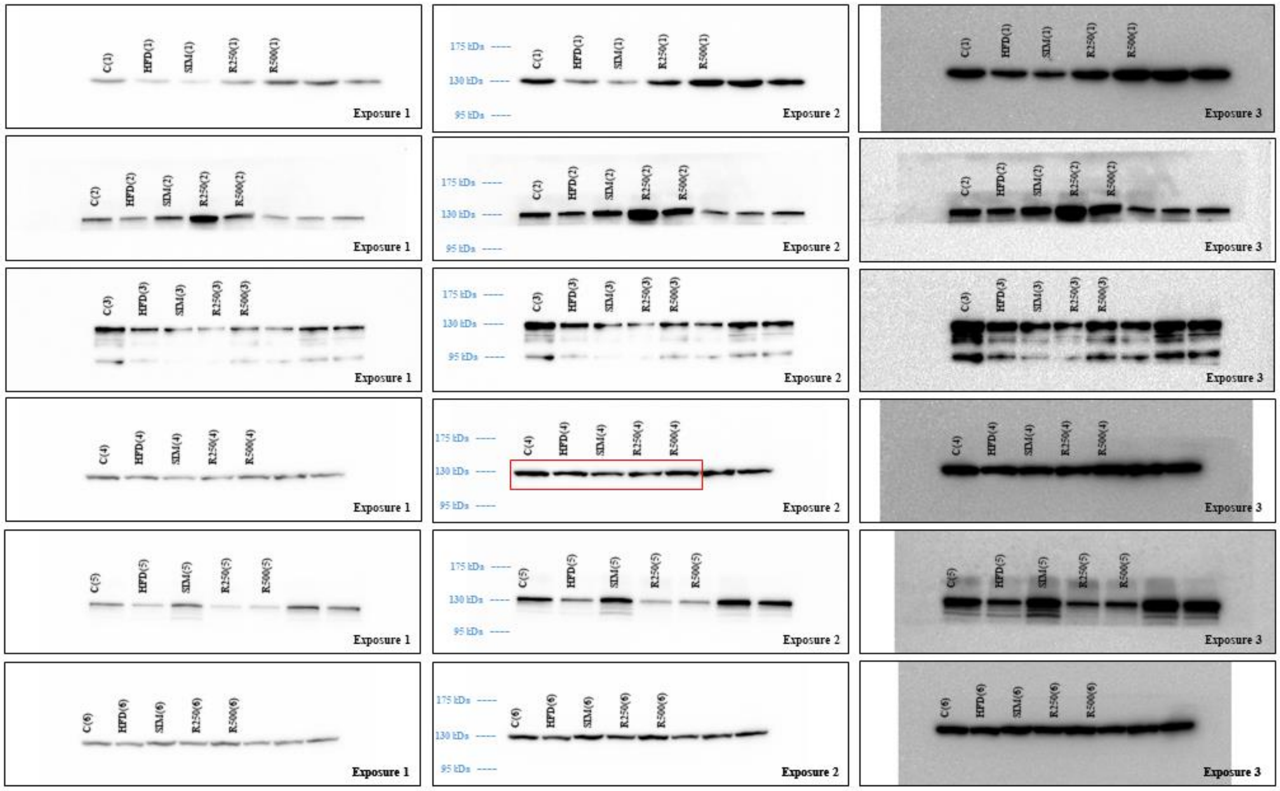

Supplementary Figure 3D: Original (uncropped) images of western blotting membranes for Figure 5A (IRS-1). Cropped blot shown in Figure 5A (IRS-1) is indicated by the framed region. The blots of repeated data with difference exposure times were shown in the supplementary figure (exposure 1, 2, and 3 at 30, 120, and 300 sec, respectively). The livers of each group were analyzed with antibodies against IRS-1 on Western blot and normalized with β-actin. The data are used to present the average of 6 individual rats ± SD (n=6 per group). The statistical analysis was performed with one-way ANOVA followed by Tukey's *post hoc* test.

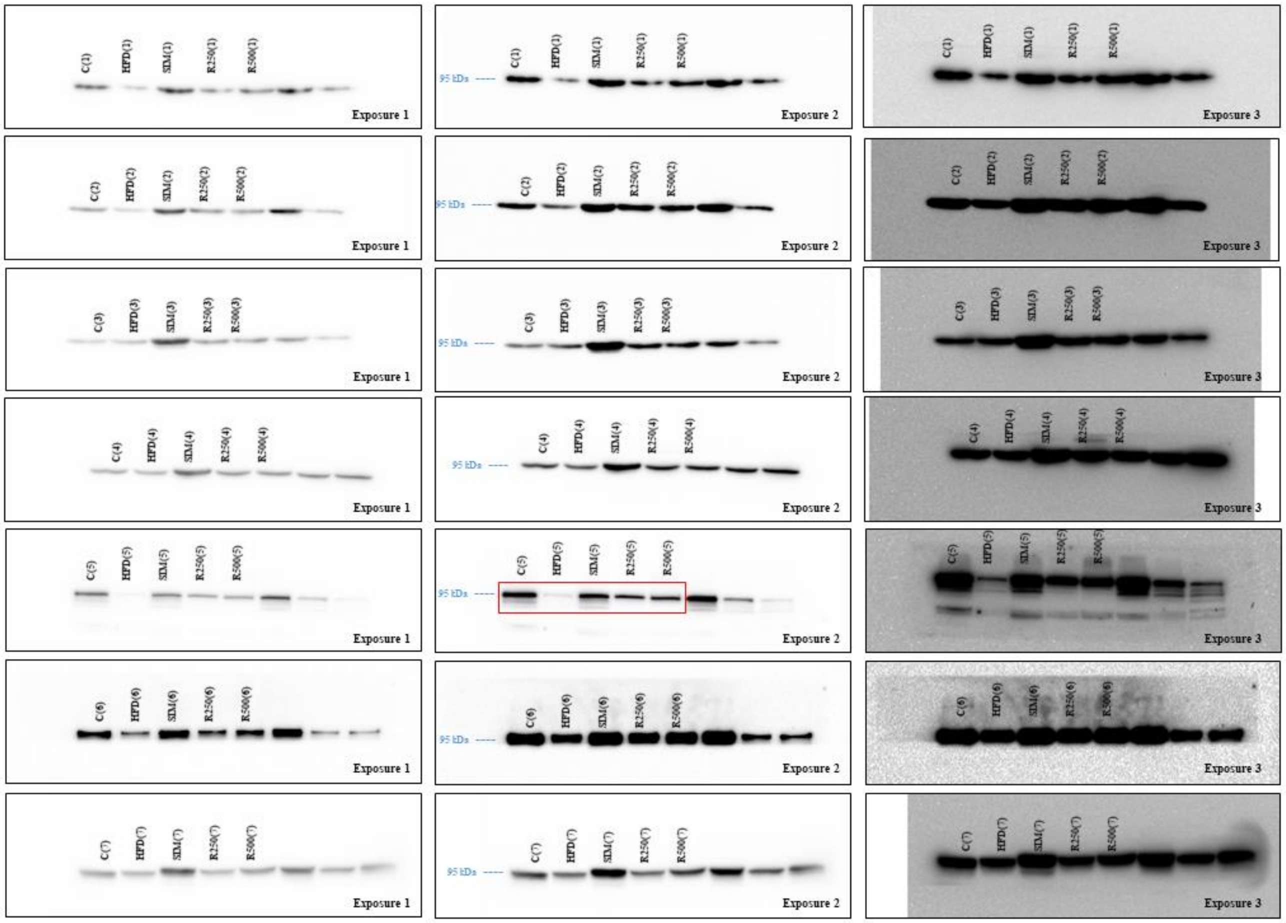

Supplementary Figure 3E: Original (uncropped) images of western blotting membranes for Figure 5A (Nrf2). Cropped blot shown in Figure 5A (Nrf2) is indicated by the framed region. The blots of repeated data with difference exposure times were shown in the supplementary figure (exposure 1, 2, and 3 at 30, 120, and 300 sec, respectively). The livers of each group were analyzed with antibodies against Nrf2 on Western blot and normalized with β-actin. The data are used to present the average of 7 individual rats ± SD (n=7 per group). The statistical analysis was performed with one-way ANOVA followed by Tukey’s *post hoc* test.

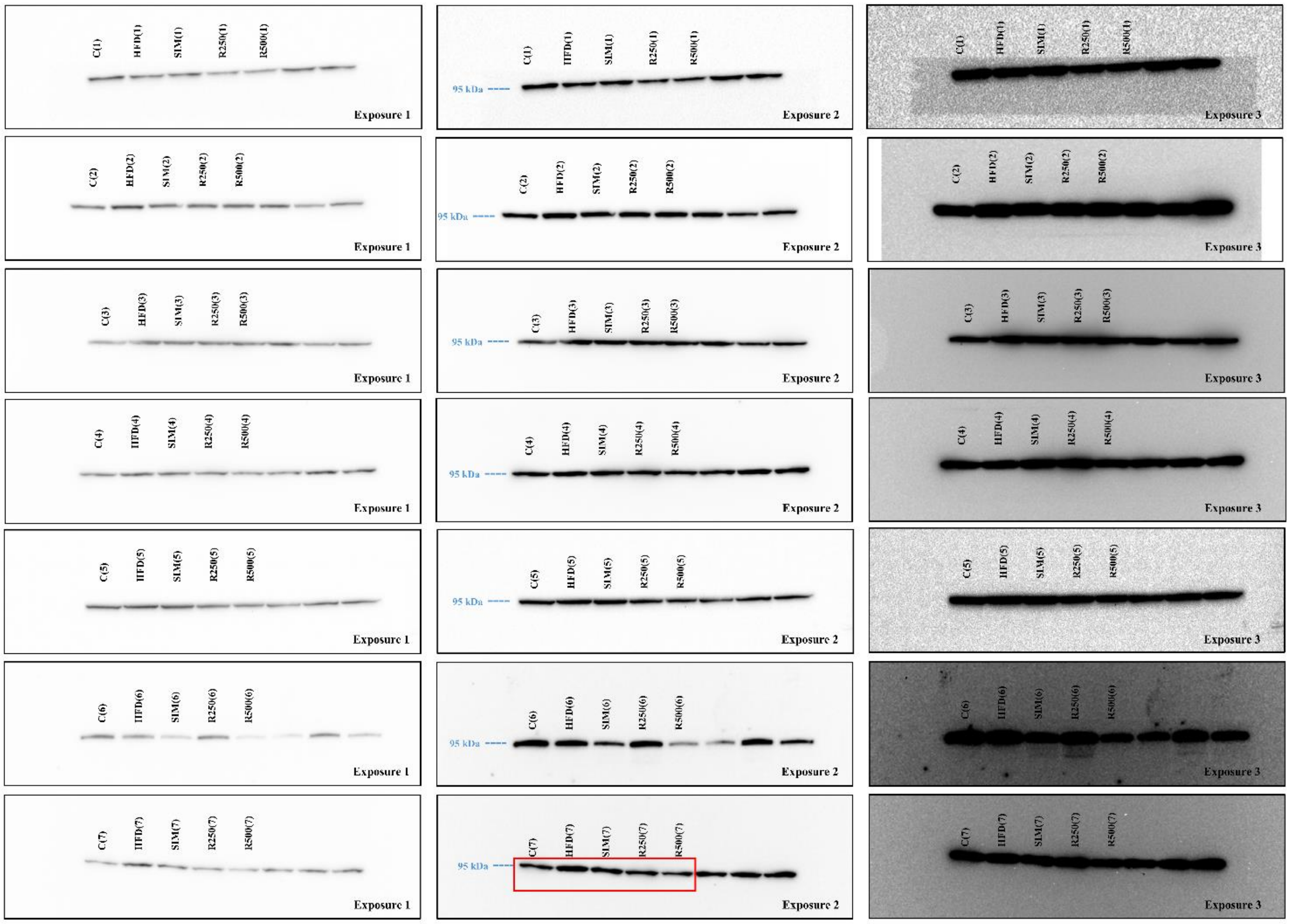

Supplementary Figure 3F: Original (uncropped) images of western blotting membranes for Figure 5A (MTP). Cropped blot shown in Figure 5A (MTP) is indicated by the framed region. The blots of repeated data with difference exposure times were shown in the supplementary figure (exposure 1, 2, and 3 at 30, 120, and 300 sec, respectively). The livers of each group were analyzed with antibodies against MTP on Western blot and normalized with β-actin. The data are used to present the average of 7 individual rats ± SD (n=7 per group). The statistical analysis was performed with one-way ANOVA followed by Tukey's *post hoc* test.

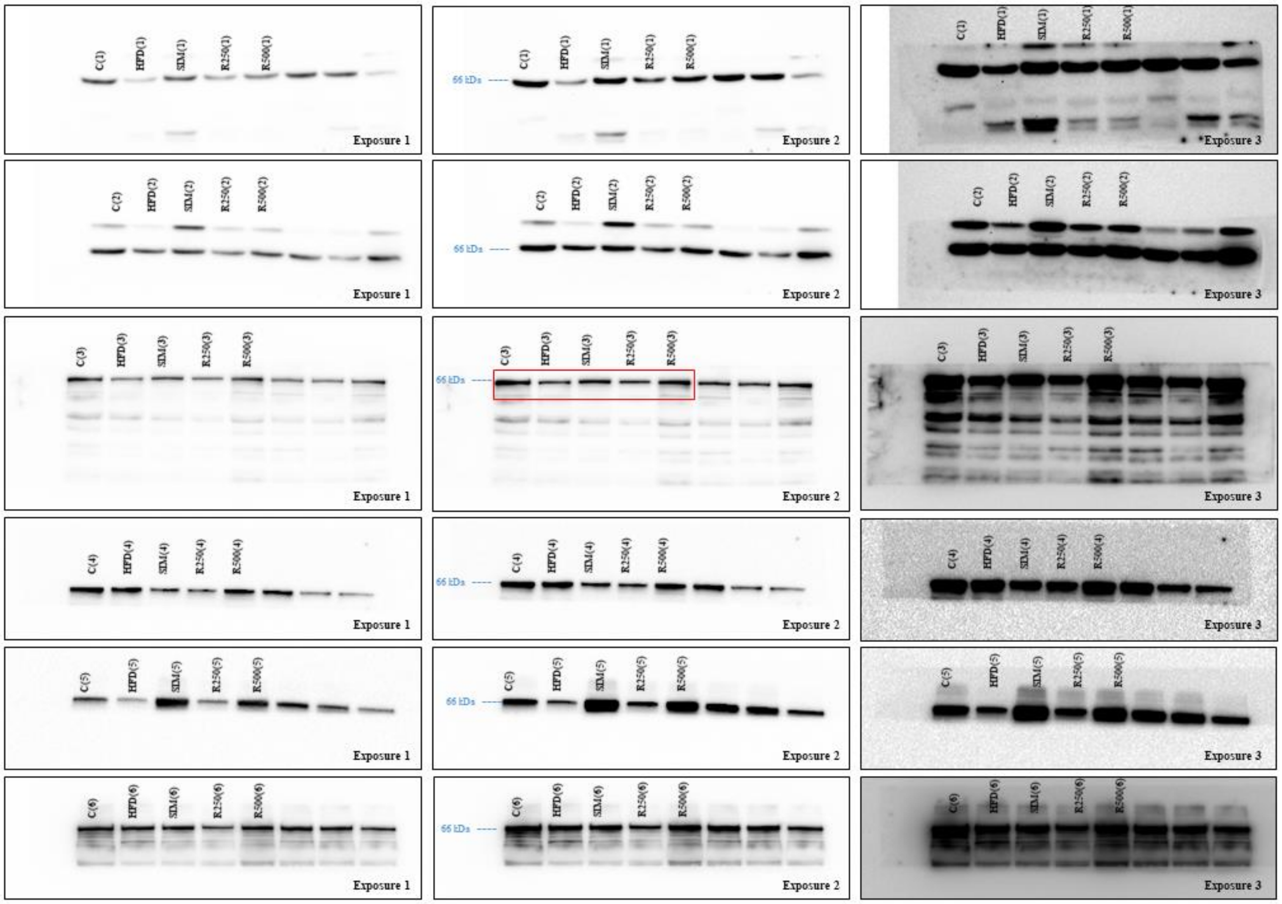

Supplementary Figure 3G: Original (uncropped) images of western blotting membranes for Figure 5A (p-Akt). Cropped blot shown in Figure 5A (p-Akt) is indicated by the framed region. The blots of repeated data with difference exposure times were shown in the supplementary figure (exposure 1, 2, and 3 at 30, 120, and 300 sec, respectively). The livers of each group were analyzed with antibodies against phospho-Akt (Ser473) on Western blot and normalized with β-actin. The data are used to present the average of 6 individual rats ± SD (n=6 per group). The statistical analysis was performed with one-way ANOVA followed by Tukey's *post hoc* test.

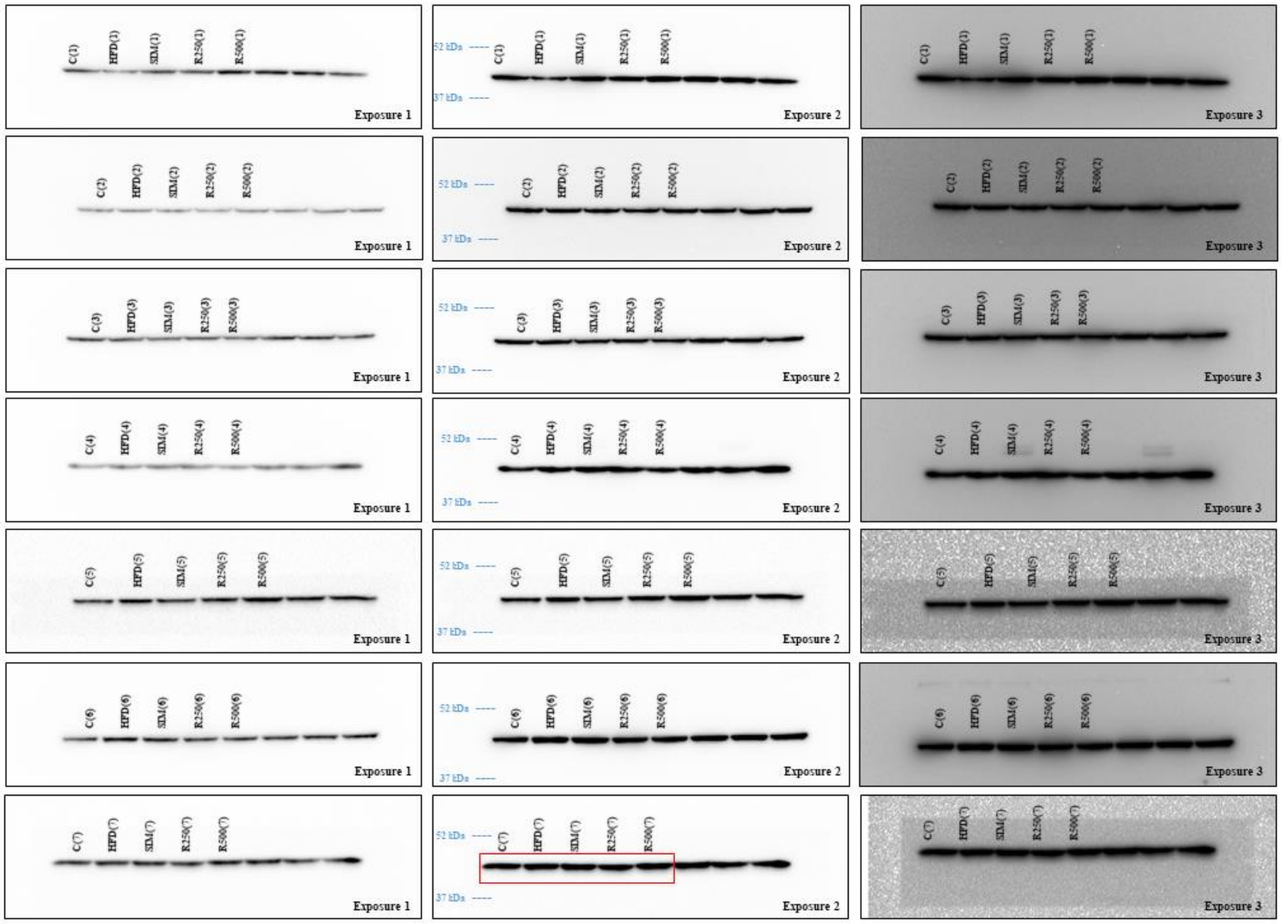

Supplementary Figure 3H: Original (uncropped) images of western blotting membranes for Figure 5A (β-actin). Cropped blot shown in Figure 5A (β-actin) is indicated by the framed region. The blots of repeated data with difference exposure times were shown in the supplementary figure (exposure 1, 2, and 3 at 30, 120, and 300 sec, respectively). The livers of each group were analyzed with antibodies against β-actin on Western blot. Representative blots were obtained from 7 individual rats.
